# Studying Development of Psychopathology Using Changing Measures to Account for Heterotypic Continuity

**DOI:** 10.1016/j.jaacop.2025.10.008

**Published:** 2025-11-07

**Authors:** Isaac T. Petersen, Zachary Demko, Won-Chan Lee, Jacob J. Oleson

**Affiliations:** a University of Iowa, Iowa City, Iowa

**Keywords:** development of psychopathology, heterotypic continuity, changing measures, developmental scaling, longitudinal

## Abstract

**Objective:**

Psychopathology shows changes in behavioral manifestation across development, that is, heterotypic continuity. However, research has paid little attention to how to account for heterotypic continuity when examining the development of psychopathology. This longitudinal study accounted for heterotypic continuity of multiple psychopathology dimensions by using developmental scaling to place multi-informant ratings of children’s behavior problems onto the same scale to chart children’s trajectories.

**Method:**

The study examined children’s (N = 231) development of 3 psychopathology dimensions—externalizing, internalizing, and thought-disordered—using different measures across 7 timepoints from 3 to 7.5 years of age. Psychopathology dimensions were assessed by mother-, father-, and teacher/caregiver-report. We compared 3 assessment approaches: the common items, upward/downward extension, and construct-valid items approaches. We compared 2 scoring approaches: mean scoring and developmental scaling. Developmental scaling aims to place scores from age-differing measures onto the same scale. We compared their accuracy, for externalizing problems, in terms of criterion validity with respect to observations of compliance and attention to task.

**Results:**

Using different measures across ages (ie, construct-valid items approach) was the most accurate assessment approach—modestly more accurate than using the common items or upward/downward extension—in terms of criterion validity with respect to observations of compliance and attention to task (*r*_diff_ = 0.07-0.13). Developmental scaling was the most accurate scoring approach, modestly more accurate than average scores (*r*_diff_ = 0.03-0.17).

**Conclusion:**

Using (1) age-differing measures to account for heterotypic continuity and (2) developmental scaling to link scores from the different measures onto the same scale may enable studying development of psychopathology across the lifespan.

**Diversity & Inclusion Statement:**

We worked to ensure sex and gender balance in the recruitment of human participants. We worked to ensure race, ethnic, and/or other types of diversity in the recruitment of human participants. We worked to ensure that the study questionnaires were prepared in an inclusive way.

**Study registration information:**

School readiness study: https://osf.io/jzxb8

## Plain language summary

Behavioral health problems manifest differently as children age. This study examined manifestations of 3 dimensions of behavioral health problems from ages 3 to 7.5 years (N = 231). The authors found that using different measures and scaling across ages was the most accurate way to assess behavioral health problems as children age.

## Graphical abstract

**Figure:**
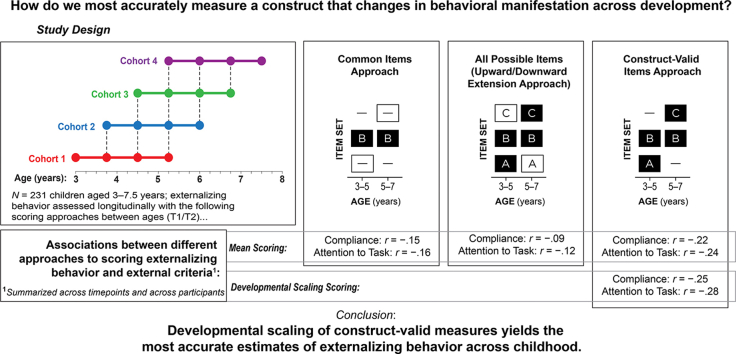

Psychopathology is thought to show heterotypic continuity—its behavioral manifestations change across development despite persistence in the underlying construct.^1^ For instance, externalizing problems—which encompass disinhibition and antagonism—are often expressed as overt acts in early childhood, such as physical aggression; however, externalizing problems are more often expressed later in development as covert and indirect or relational forms of aggression, rule breaking, and substance use.^2^ These behaviors are often considered covert or indirect because, unlike overt physical aggression, they are less visible, less confrontational, or occur outside the immediate view of authority figures or of individuals targeted by the aggressive behavior. A consequence of the changing behavioral manifestation of psychopathology is that different measures across development—and methods to link their scores onto the same scale—may be necessary to chart children’s development of psychopathology while accounting for heterotypic continuity. Despite considerable evidence^3^ and theory^4^ demonstrating that psychopathology shows changes in manifestation across development, heterotypic continuity has largely been ignored when studying people’s trajectories. Nearly all longitudinal studies that chart people’s development of psychopathology use the same measure across time,^1^ likely leading to inaccurate trajectories.^5^, ^6^, ^7^, ^8^ Moreover, emerging conceptual frameworks of psychopathology, including the Hierarchical Taxonomy of Psychopathology (HiTOP) and Research Domain Criteria (RDoC), do not account for development.^9^, ^10^, ^11^ Thus, developing approaches to account for heterotypic continuity represents a critical challenge for advancing understanding of how psychopathology develops.

It is important to examine continuity and discontinuity across key developmental transitions, such as the transition from preschool to school entry. Entry into formal schooling often introduces structured demands (eg, sitting still and completing tasks) and additional socialization agents, including teachers and a transition from a reliance on caregivers to closer affiliation with peers. Although heterotypic continuity has since been demonstrated across the lifespan,^3^ Kagan argued that heterotypic continuity is particularly likely before age 10—during which time children learn more effective ways of accomplishing their goals^12^ and develop skills in inhibiting inappropriate actions^13^—and after major changes in their psychological ecology, including transition to school.^13^ Some behaviors likely emerge across this developmental period, such as lying, supported by developing skills in theory of mind and executive functioning,^14^ whereas other behaviors may wane with age, such as biting others and having temper tantrums. Determining how to chart individuals’ change across the transition from preschool to school entry is a necessary step toward the goal of tracking people’s change across the lifespan. The present study examines children’s development of multiple dimensions of psychopathology across 3 to 7.5 years of age during this crucial transition from preschool to school entry, and examines the accuracy of various approaches to longitudinal assessment of externalizing behavior.

There are 2 primary ways that prior work has examined people’s change in psychopathology across development (Figure 1).^6^ The “common items” approach removes items that are age specific. For example, in a study of externalizing problems from early to middle childhood, a study might remove some physical aggression items such as “bites others” that might not be developmentally relevant at all ages. The second approach, the “upward/downward extension” approach, takes items that are valid at a given age and uses the items across ages when the item may not be valid or useful; that is, all items are assessed at all ages. For instance, the upward extension approach might take the item “disobedient to authority figures” and apply it to older individuals for whom such a behavior may no longer validly reflect externalizing behavior and may instead reflect prosocial functions including protesting against societally unjust actions.^6^ Both the “common items”^15^^,^^16^ and “upward/downward extension”^17^, ^18^, ^19^ approaches are widely used, likely because they result in the same items assessed across ages; however, both have key problems. As an example of the common items approach, Odgers *et al.*^15^ examined children’s development of *DSM-IV* symptoms of conduct disorder from ages 7 to 26 years, but they dropped age-specific symptoms (eg, running away, staying out late) “because [these symptoms] did not cover the study’s age span.”^p676^ As an example of the upward extension approach, Broeren *et al.*^18^ examined children’s development of anxiety from ages 4 to 11 years; they noted, “Although the questionnaire was originally developed to measure anxiety in preschoolers, the current study also employed the scale with older children to promote uniformity in measures.”^p84^ Forcing use of the same measure across time naturally limits the ages that a study can span, which prevents charting wider age spans. For example, one could not examine development of depression across ages 11 to 13 years using the Short Mood and Feelings Questionnaire because of changes in its functioning (in particular, some items were less relevant at age 11) that likely reflect developmental changes in manifestation of depression.^20^ The common items approach yields low content validity because it does not assess all construct facets, especially age-specific manifestations. The upward/downward extension approach violates construct validity because it assesses developmentally inappropriate items. In sum, despite these approaches being the most widely used for assessing individuals’ development, they likely lead to inaccurate scores and thus inaccurate trajectories.

**Figure 1:**
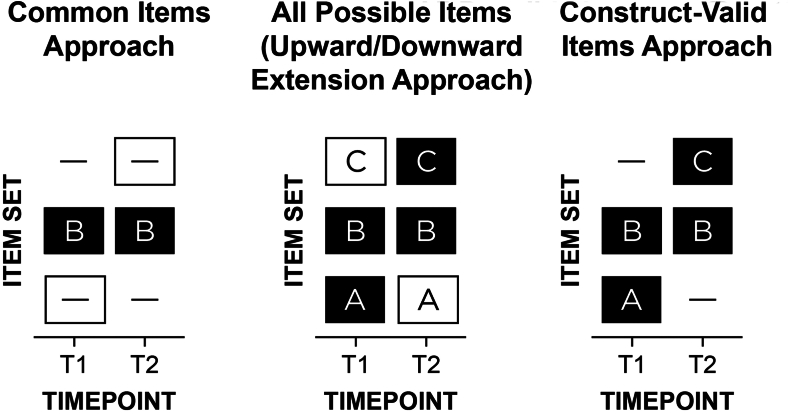
Approaches to Longitudinal Assessment ***Note:****Item set A refers to items that are construct-valid at only timepoint 1 (T1); item set B is construct-valid at both T1 and T2; item set C is construct-valid at only T2. A dash indicates that the item set was not assessed at a given timepoint. A white box indicates invalid assessment in terms of either a content gap (ie, important missing items) or intrusion (ie, invalid items at a given timepoint). In a study of externalizing problems from early childhood to adulthood, “biting others” may be in item set A; “noncompliant” in B; “drug use” in C. The 3 approaches are as follows: (1) common items: B at T1 and T2; (2) upward/downward extension: ABC at T1 and T2; or (3) construct-valid items: AB at T1; BC at T2. The “common items” and “upward/downward extension” approaches are by far the most widely used in the literature, even though they likely lead to inaccurate scores and thus inaccurate trajectories. In the present study, the change in measurement occurred between ages 5 and 6 years (ie, the Child Behavior Checklist 1.5–5 and Caregiver–Teacher Report Form were assessed from ages 3 to 5 1/4 years; the Child Behavior Checklist 6–18 and Teacher’s Report Form were assessed from ages 6 to 7.5 years).*

The present study considers a third approach to assess individuals’ development of psychopathology: the “construct-valid items” approach (Figure 1). The construct-valid items approach uses different items, as needed, across time to maintain construct validity and to account for the changing behavioral manifestation of psychopathology. Because the construct-valid items approach uses different measures across development when the construct changes in manifestation, scoring approaches are needed to link scores from the age-differing measures onto the same scale in ways that allow tracking individuals’ absolute growth. Traditional scoring approaches (eg, mean, sum) do not ensure that scores from the differing measures are comparable, and age-normed scores do not allow observing absolute growth. A potentially useful scoring approach for linking scores from age-differing measures onto the same scale is developmental scaling.^6^ Developmental scaling (aka vertical scaling) refers to the process of linking or harmonizing scores from different measures across development onto the same scale while retaining absolute change (unlike age-normed scores).^21^ There are various approaches to developmental scaling such as item response theory–based approaches. The present study performs developmental scaling as a function of age (ie, age-based scaling). Developmental scaling benefits from having some age-common items from measures at adjacent ages, to serve as an anchor. Developmental scaling can leverage the common items to link the scores from the different measures onto the same scale (ie, the scale of the reference age). By assuming no changes in functioning of the common items across ages (ie, no differential item functioning) or by accounting for any changes in item functioning, this approach ensures that both item and latent parameter estimates are expressed on a common scale across ages. Nevertheless, developmental scaling can use all construct-valid items—both common and unique items—to estimate people’s scores on that scale, thus making use of all construct-valid information while estimating people’s scores on a comparable scale across development.

Simulation work has demonstrated that, compared to the common items and upward/downward extension approaches, the construct-valid items approach yields more accurate trajectories at the group and person level.^7^ Although many studies have examined development of psychopathology, few studies have examined trajectories in ways that account for heterotypic continuity, that is, by using different, age-appropriate measures across time to maintain construct validity,^22^^,^^23^ which is crucial for accurate inferences. Even fewer have done so in ways that allow identifying absolute change, which is necessary to chart individual development. We are aware of only 3 studies that have used the construct-valid items approach (ie, age-differing measures) to study individuals’ absolute change in psychopathology,^8^^,^^24^^,^^25^ in the case of externalizing problems^24^^,^^25^ and internalizing problems.^8^^,^^24^ This is surprising, because the use of age-differing measures is common when studying individuals’ development in other domains, including cognitive and educational development.^26^, ^27^, ^28^ Moreover, no studies have performed developmental scaling of the 3 primary dimensions of psychopathology simultaneously—including the externalizing, internalizing, and thought-disordered dimensions of psychopathology.^29^ Externalizing problems encompass disinhibition and antagonism; internalizing problems encompass mood and anxiety problems; thought-disordered problems encompass psychosis. Collectively, externalizing, internalizing, and thought-disordered problems are thought to parsimoniously describe many forms of psychopathology.^29^ Moreover, the 3 dimensions covary, so there is value in modeling them together. In addition, despite theory and simulation work supporting the construct-valid items approach,^7^ no studies have compared the common items, upward/downward extension, and construct-valid items approaches empirically.

### The Present Study

The present study demonstrates and evaluates the use of the following: (1) different yet construct-valid items across time—ie, the “construct-valid items” approach—to account for the changing behavioral manifestation of psychopathology across development; and (b) developmental scaling methods to place the scores from the different items onto the same scale to allow charting of individuals’ development. Consistent with modern conceptualizations of the (multi)dimensionality of psychopathology, we examine the development of externalizing, internalizing, and thought-disordered dimensions of psychopathology during childhood. The present study is the first to use different measures of externalizing, internalizing, and thought-disordered psychopathology over time (and we leverage overlapping items) while using developmental scaling to link scores from the measures onto the same scale for more accurate growth estimates that account for heterotypic continuity. This allows us to chart children’s development in externalizing, internalizing, and thought-disordered psychopathology across a lengthy age span despite changes in behavioral manifestation and measurement.

We test 3 research questions (RQs), as follows:

RQ1: What is the developmental course of externalizing, internalizing, and thought-disordered psychopathology for boys and girls across 3 to 7 years of age? To examine this question, we examine growth curves of developmentally scaled scores that leverage the construct-valid items.

RQ2: Which approach to longitudinal assessment is most accurate using mean scoring: the common items, upward/downward extension, or construct-valid items approach? To examine this question, we compare their criterion validity for estimating externalizing problems by examining their strength of association with researcher observations of children’s externalizing behavior, including (non)compliance and (in)attention to task.

RQ3: Is developmental scaling of construct-valid items on different measures across time an accurate and useful way to place scores from different measures onto the same scale and account for heterotypic continuity? We compare the accuracy (criterion validity) of developmental scaling using different measures across time (ie, construct-valid items) to traditional approaches (ie, mean scoring of the common items and upward/downward extension approaches) that use the same items across time and ignore heterotypic continuity. We also evaluate whether developmentally scaled scores of externalizing problems show usefulness (incremental validity) in predicting the observations of noncompliance and inattention over and above predictions from traditional approaches. In addition, we evaluate traditional approaches’ misclassification of individuals’ persistence vs desistance of behavior problems.

We hypothesized that psychopathology undergoes changes in behavioral manifestation across development, consistent with heterotypic continuity; and consequently, that to accurately assess children’s development of psychopathology, it is necessary to use age-differing measures with approaches that link the scores from the different measures to be on the same scale. If this hypothesis is true, we predict the following: (1) the construct-valid items approach to assessment will show greater accuracy (ie, stronger criterion validity) than the common items and upward/downward extension approaches; and (2) developmental scaling of the construct-valid items will show incremental validity above and beyond the common items and upward/downward extension approaches. Our hypotheses were preregistered (https://osf.io/jzxb8).

## Method

### Participants

A community sample of children (N = 231) and their caregivers participated in an ongoing accelerated longitudinal study. Participants were recruited at 1 of 4 ages: 36 (n = 62), 45 (n = 54), 54 (n = 53), or 63 (n = 62) months. Spanning all timepoints, children ranged from 3 to 7.5 years of age. The inclusion criterion to be recruited for the study was that the child was one of the target ages (described above). Exclusion criteria were as follows: the child’s primary caregiver did not speak English, or the child did not have a permanent guardian, did not have normal or corrected-to-normal vision and hearing, or was not capable of communicating or following basic instructions in English. Participants were recruited in 2018 to 2024 through a biomedical registry of children who had well-child checkups at the University of Iowa Health Care Medical Center, from university listservs, and from advertisements and in-person recruitment activities at local schools, daycare facilities, and preschools, Women, Infants, and Children (WIC) programs, pediatricians’ offices, and community events; and by word of mouth. Reasons for participant ineligibility and a flowchart of the final sample are shown in Figure S1, available online. Extent of missingness and tests of systematic missingness are in Supplement 1, available online. The sample consisted of children, their primary caregiver, the primary caregiver’s parenting partner (as applicable), and a teacher/secondary caregiver (eg, nanny, babysitter, or someone else who knew the child well). Participant demographics are listed in Table 1. Compared to the US population, sample participants were somewhat more likely to be White, married, middle or upper class, and to have a college or graduate degree. Participant demographics were broadly reflective of the surrounding area.

**Table 1:** Participant Demographics

	n/Mean	%/SD
Child		
Sex		
Male	122	52.8
Female	109	47.2
Age, y	4.96	1.19
Ethnicity		
Hispanic	38	16.5
Not Hispanic	193	83.5
Race		
Asian	9	3.9
Black or African American	16	6.9
White	170	73.6
More than 1 race	24	10.4
Other race	12	5.2
Primary caregiver		
Sex		
Male	21	9.1
Female	210	90.9
Age, y	35.36	5.31
Education		
Some high school (no degree)	6	2.6
High school degree	8	3.5
Some college	30	13.1
Associate’s degree	28	12.2
Bachelor’s degree	80	34.9
Master’s degree	57	24.9
Professional school degree	8	3.5
Doctorate degree	12	5.2
Marital status		
Single/never married	29	12.7
Married	184	80.3
Separated	6	2.6
Divorced	8	3.5
Re-married	2	0.9
Parenting partner		
Sex		
Male	197	86.0
Female	32	14.0
Age, y	37.24	6.48
Education		
No schooling (or <1 y)	1	0.4
Nursery, kindergarten, or elementary (grades 1-8)	1	0.4
Some high school (no degree)	9	4.0
High school degree	25	11.2
Some college	40	17.9
Associate’s degree	29	13.0
Bachelor’s degree	61	27.4
Master’s degree	35	15.7
Professional school degree	12	5.4
Doctorate degree	10	4.5
Family		
Income-to-needs ratio	3.53 (median = 3.05)	2.84
Hollingshead Four-Factor Index of Socioeconomic Status	47.9 (possible range: 1-66)	12.4
Nam–Powers–Boyd Occupation Status score, averaged across parents	71.2 (possible range: 0-100)	20.6
Study variables		
Externalizing problems (developmentally scaled)^a^	−0.77	2.67
Internalizing problems (developmentally scaled)^a^	−1.63	2.86
Thought-disordered problems (developmentally scaled)^a^	−2.05	2.96
Externalizing problems (construct-valid items)^b^	0.15	0.14
Externalizing problems (common items)^b^	0.14	0.15
Externalizing problems (all possible items; upward/downward extension approach)^b^	0.15	0.13
Externalizing problems (*T* score)	47.11	9.79
Internalizing problems (*T* score)	45.69	9.51
Attention to task (observation)	3.93 (possible range: 1-5)	0.96
Compliance (observation)	4.08 (possible range: 1-5)	0.99

Note: For some variables, the number of primary caregivers and parenting partners is greater than the number of children because the primary caregiver and parenting partner in some cases changed over time. The sample-wide mean of the developmentally scaled scores is negative and the SD is greater than 1.0, indicating that behavior problems (across ages) tended to be lower than the latent level at age 3 years and that scores tended to show greater variability relative to the variability at age 3.

a Scores are on the scale of the factor scores at 3 years of age.

b Computed with a mean (proportion) score.

### Procedures

The child and their primary caregiver (ie, parent) participated in 2 laboratory visits every 9 months for 4 timepoints (Figure S2, available online). The first laboratory visit consisted of parent–child interaction tasks and behavioral tasks; it lasted ∼2.5 hours. During the behavioral tasks, the parent completed questionnaires on their child. The second laboratory visit consisted of the child completing computerized tasks while electroencephalography was recorded; it lasted ∼2 hours. The primary caregiver’s parenting partner (as applicable) and a teacher/secondary caregiver were invited to complete electronic questionnaires on the child. Video examples of procedures are available on Databrary (https://nyu.databrary.org/volume/1559).

### Measures

The present study is part of a larger study, the School Readiness Study. Measures and hypotheses for the School Readiness Study were preregistered (https://osf.io/jzxb8). Data files, a data dictionary, analysis scripts, and a computational notebook for the present study are published online (https://osf.io/bgxma).

#### Behavior Ratings of the Child’s Psychopathology

Children’s psychopathology was rated by mothers, fathers, and teachers/secondary caregivers. Mothers and fathers completed the Child Behavior Checklist (CBCL) 1.5–5^30^ or CBCL 6–18,^31^ depending on the child’s age. Teachers/secondary caregivers completed the Caregiver–Teacher Report Form (C–TRF)^30^ or Teacher’s Report Form (TRF),^31^ depending on the child’s age. Items were on a scale of 0 to 2, in which 0 = “Not true (as far as you know)”; 1 = “Somewhat or sometimes true”; 2 = “Very true or often true.” Externalizing items were those from the Externalizing scale, which includes the Attention Problems and Aggressive Behavior subscales for the CBCL 1.5–5 and C–TRF; it includes the Rule-Breaking Behavior and Aggressive Behavior subscales for the CBCL 6–18 and TRF. Internalizing items were those from the Internalizing scale, which includes the Emotionally Reactive, Anxious/Depressed, Somatic Complaints, and Withdrawn subscales for the CBCL 1.5–5 and C–TRF; it includes the Anxious/Depressed, Withdrawn/Depressed, and Somatic Complaints subscales for the CBCL 6–18 and TRF. Thought disorder items were those from the Autism Spectrum Problems *DSM*-oriented scale for the CBCL 1.5–5 and C–TRF; thought disorder items were from the Thought Problems subscale for the CBCL 6–18 and TRF.

Externalizing and internalizing problems showed strong internal consistency (Table S1, available online), whereas thought-disordered problems showed weaker internal consistency. Behavior problem ratings showed cross-rater reliability (Table S2, available online) and cross-time rank-order stability (Table S3, available online). Cross-informant associations ranged from modest to large, consistent with prior work.^32^^,^^33^ Using age and sex norm-referenced *T* scores of 65 as a clinical cutoff, ∼12.6% and ∼7.8% of children were in the at-risk or clinical range for externalizing problems and internalizing problems, respectively, at one or more timepoints based on ratings from one or more raters.

The Achenbach scales (CBCL 1.5–5, CBCL 6–18, TRF, C–TRF) have some items that are common across all instruments (ie, common items), and some items that are unique to a particular instrument (ie, unique items). The number of common items for each pair of measures is provided in Table 2. To examine effects of upward/downward extension, we assessed both the age-common and age-unique items of each of the CBCL Externalizing scales at all ages. That is, we assessed all items from the parent-reported Externalizing scales of the CBCL 1.5–5 and CBCL 6–18 at all ages (including the non–age-relevant items). Thus, our study is uniquely positioned to determine which approach is most accurate: (1) the “common items” approach, (2) the “upward/downward extension” approach, or (3) the “construct-valid items” approach (Figure 1). To minimize teachers’ response burden, teachers did not complete non–age-relevant versions (eg, teachers of 6-year-olds did not complete the C–TRF). Given differing numbers of items and consistent with recommendations,^34^ scores for the (non–developmentally scaled) scores were calculated by averaging scores across items and dividing by 2 (the maximum possible score for a given item), thus representing a proportion of the maximum possible score. Developmentally scaled scores were estimated using a Bayesian longitudinal item response model (described later).

**Table 2:** Number of Common Items for Each Pair of Measures

Externalizing problems				
Measure	CBCL 1.5–5	CBCL 6–18	C–TRF	TRF
CBCL 1.5–5	24			
CBCL 6–18	7	35		
C–TRF	24	10	34	
TRF	9	28	12	32

Internalizing problems				
Measure	CBCL 1.5–5	CBCL 6–18	C–TRF	TRF
CBCL 1.5–5	36			
CBCL 6–18	12	32		
C–TRF	30	11	32	
TRF	11	30	11	33

Thought-disordered problems				
Measure	CBCL 1.5–5	CBCL 6–18	C–TRF	TRF
CBCL 1.5–5	12			
CBCL 6–18	1	15		
C–TRF	12	1	12	
TRF	1	10	1	10

Note: The upper third of the table presents the number of common items on the Externalizing scale. The middle third of the table presents the number of common items on the Internalizing scale. The lower third of the table presents the number of common thought-disordered problem items. Numbers on the diagonal represent the total number of items in the Externalizing scale (upper), Internalizing scale (middle), or thought-disordered problem items (lower) for that measure (eg, the CBCL 1.5–5 has 24 items on the Externalizing scale, 36 items on the Internalizing scale, and 12 thought-disordered problem items). Numbers below the diagonal represent, for that pair of measures, the number of items that are common to both of the measures. The number of unique items can be calculated by subtracting the number of common items from the total number of items. For instance, the CBCL 6–18 has 7 unique externalizing items when compared with the TRF (ie, 35 total items minus 28 common items). Conversely, the TRF has 3 unique externalizing items when compared with the CBCL 6–18 (ie, 33 total items minus 30 common items). CBCL = Child Behavior Checklist; C–TRF = Caregiver–Teacher Report Form; TRF = Teacher’s Report Form.

#### Researcher Observations of the Child’s Behavior Problems

Trained researchers observed the child’s behavior problems live during 2 laboratory visits at each timepoint. Researchers observed the child’s global degree of compliance, attention to task, and distress across the duration of each of the 2 laboratory visits. Researchers responded to the following question on a form at the end of each laboratory visit, which was designed specifically for this study: “Rate the target child in the following categories based on the entirety of the laboratory visit (1 = very low, 5 = very high): attention, compliance, distress.” The present study focused on the observations of compliance and attention to laboratory visit tasks, given their relevance for externalizing psychopathology—namely, the inattention and oppositionality aspects of externalizing behavior. As such, each rating reflected observations of child compliance and attention across a range of tasks over the span of 2 to 3 hours, such as executive function and delay-of-gratification tasks during the first laboratory visit, and inhibitory control and attentional orienting tasks while wearing an electroencephalography cap during the second laboratory visit. We did not prompt observers to consider specific behaviors when forming their judgment about their ratings. One example of a behavior that observers considered inattentive was interrupting tasks with unrelated comments; one example of a behavior that observers considered noncompliant was moving around excessively when asked to sit still.

Three researchers provided observation ratings at the end of the first laboratory visit; 2 researchers provided observation ratings at the end of the second laboratory visit. In total, at a given timepoint, 5 observers provided ratings based on ∼5 hours of laboratory visits. Each researcher’s ratings were made masked to the ratings by the other observers. Such post-visit researcher direct observation ratings are commonly used in developmental research^35^ and have been found to be associated with parent ratings and task-based coder ratings of the same behaviors in prior studies.^36^ Interobserver reliability was intracorrelation coefficient (ICC)[2,*k*] = 0 .93 and 0.85 for compliance at the first and second laboratory visit, respectively. Interobserver reliability was ICC[2,*k*] = 0.91 and 0.84 for attention to task at the first and second laboratory visit, respectively. Correlations of ratings across the first and second laboratory visits were *r* = 0.60 for compliance (*p* values <.001) and *r* = 0.67 for attention to task. Ratings were first averaged across raters within visit, and then were averaged across visits within timepoints.

### Statistical Analysis

We used developmental scaling to link scores from the construct-valid items, from different measures, across ages and raters onto the same scale (Supplement 2, available online). The item response theory (IRT) approach to developmental scaling places scores from different measures onto the same scale by linking the item response theory parameters of the common items (ie, easiness and discrimination) to ensure that the scores are comparable across ages and raters. We used a 2-parameter Bayesian longitudinal item response model in a mixed modeling item response theory framework that simultaneously does the following: (1) performs developmental scaling to link the scores from the differing measures onto the same scale, (2) estimates children’s growth curves of psychopathology, and (3) accounts for potential differential item functioning across ages and raters. This allowed charting children’s development of psychopathology on a comparable scale across ages 3 to 7 years, despite measurement changes. We included a quadratic trend to allow curvature in children’s trajectories over time. Age in years was centered to set intercepts at age 3 years, the youngest age in the sample. Age 3, therefore, serves as the reference scale, with parameter estimates for subsequent time points modeled relative to this baseline. Thus, children’s factor scores (theta) were on the scale of the factor scores at 3 years of age.

In this longitudinal item response theory model, item and person parameter estimates across ages and raters were placed on a common scale using common items and simultaneous calibration. We accounted for age-related differences in item functioning of item parameters (easiness and discrimination), which ensured that the person parameter estimates at different age groups were expressed on the same scale. Rather than estimating parameters separately for each age group, all age-specific measures were calibrated simultaneously within a single model, resulting in item and person parameter estimates on the same underlying scale. To prevent arbitrary shifts in scale, the model imposed an additional constraint: the variance of the latent factor at age 3 was fixed at 1, and the mean was ∼0. The scales for subsequent ages were then determined through the common items, preserving score comparability over time.^37^^,^^38^

The 2-parameter item response model estimates 2 parameters of each item: easiness and discrimination. Easiness is the expected score on the item at a given level of the latent factor. Discrimination of an item reflects how strongly the item is associated with the latent factor. The model used a multidimensional item response theory approach to allow items to load onto the latent externalizing, internalizing, externalizing, or thought-disordered factor. This allowed borrowing information from each dimension in estimation of the others for more accurate estimates, given considerable covariation among externalizing, internalizing, and thought-disordered problems. Items on the Externalizing scale of the CBCL and (C–)TRF were allowed to load onto the latent externalizing factor. Items on the Internalizing scale of the CBCL and (C–)TRF were allowed to load onto the latent internalizing factor. Items on the Autism Spectrum Problems (CBCL 1.5–5, C–TRF) and Thought Problems (CBCL 6–18, TRF) subscales were allowed to load onto the latent thought-disordered factor. The item response theory model was a graded response model with a cumulative response distribution and a logit link, which allows ordinal responses.

We also compared criterion validity of developmentally scaled scores and traditional scoring methods including a mean (ie, proportion) score for each of the following: (1) the “common items,” (2) items from the “upward/downward extension” approach, and (3) the “construct-valid items” (ie, scoring the items used in the developmental scaling model with traditional methods). To compare their criterion validity, we used the Fisher *r*-to-*z* tests to examine their strength of association with researcher observations of children’s behavior problems (ie, low compliance and attention to task). Using Bayesian mixed-effects models, we also evaluated whether developmental scaling of the construct-valid items showed incremental validity above and beyond the traditionally scored common items and upward/downward extension approaches in predicting research observations of behavior. Mixed-effects models included random intercepts for the participant to account for nonindependence of multiple observations from the same child, given the longitudinal and multi-informant study design. Models were fit using the brms package^39^ version 2.22.0 in R^40^ version 4.3.1. Models were estimated with 4 chains and 4,000 iterations. We kept default brms priors,^39^ which use vague but proper priors.

Parents and teachers completed the items for the relevant full scale; however, only parents completed the additional items for the non–age-relevant version that are necessary for evaluating the upward/downward extension approach. Thus, for a fair comparison with the upward/downward extension approach, we used only parents’ ratings. For comparisons that did not involve the upward/downward extension approach (common items vs construct-valid items; traditional scoring vs developmentally scaling), we used both parents’ and teachers’ ratings. Tests were 2-tailed.

To distinguish individuals’ growth curves as either persisting or desisting, we conducted latent class growth analyses (Supplement 3, available online).

### Sensitivity Analysis

We conducted sensitivity analyses when imposing approximate longitudinal measurement invariance constraints in the developmental scaling model (Supplement 4, available online).

## Results

Descriptive statistics and bivariate correlations for study variables are provided in Table S4, available online. Numbers of observations at each timepoint are in Table S5, available online. Regression coefficients of the Bayesian item response model are in Table S6, available online. Item parameters by age and rater are in Table S7, available online. Estimates of changes in item functioning with age are in Table S8, available online. A description of item functioning is provided in Supplement 5, available online.

### Developmental Course of Externalizing, Internalizing, and Thought-Disordered Problems

**Figure 2:**
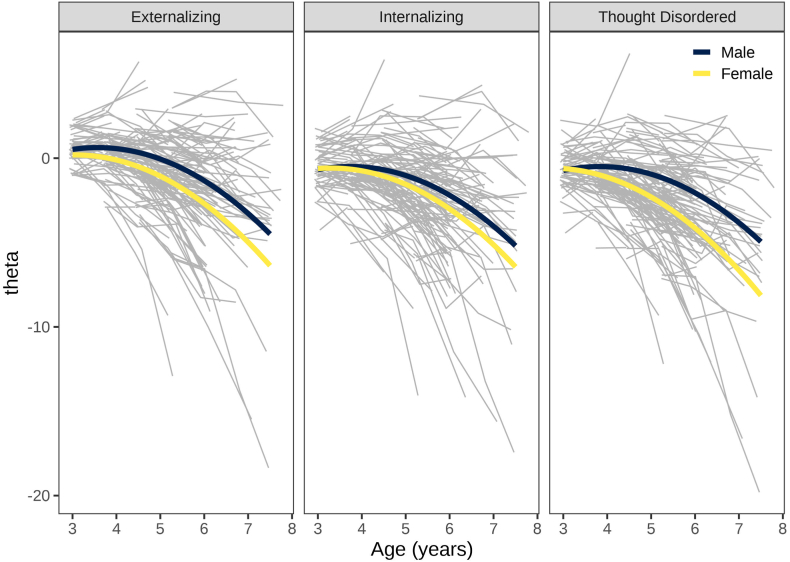
Developmentally Scaled Trajectories of Externalizing, Internalizing, and Thought-Disordered Problems ***Note:****The figure depicts participants’ model-implied values of theta overlaid with the sample’s model-implied trajectory by sex. The gray lines represent participants’ model implied values of theta by age. The yellow and dark blue lines represent the sample’s model-implied trajectory by sex. Theta represents the estimate of the person’s level on the latent factor. Theta is on the scale of the factor scores at 3 years of age. A theta of zero represents the average latent level of behavior problems at age 3; a positive theta indicates that the child is above the average latent level of behavior problems at age 3; a negative theta indicates that the child is below the average latent level of behavior problems at age 3.*

Developmentally scaled trajectories of externalizing, internalizing, and thought-disordered problems are depicted in Figure 2. On average, boys and girls showed rapid decreases in levels of psychopathology across 3 to 7 years of age. Boys tended to show higher levels than girls at the older ages, particularly in externalizing and thought-disordered problems. Girls showed steeper declines than boys in externalizing and thought-disordered problems. In latent class growth analyses, the common items and upward/downward extension approaches led to considerable misclassification of persistence vs desistance with respect to developmental scaling.

### Criterion Validity

The Fisher *r*-to-*z* tests of comparative criterion validity are shown in Table S10, available online. As expected based on theory and simulation work, average scores of externalizing problems using the construct-valid items approach showed stronger criterion validity (compliance: *r* = −0.22; attention to task: *r* = −0.24) compared to the following: (1) the common items approach (compliance: *r* = −0.15; attention to task: *r* = −0.16); (2) the upward/downward extension approach (compliance: *r* = −0.09; attention to task: *r* = −0.12); (3) age and sex norm-referenced *T* scores from the Achenbach scales; and (4) in-sample *z* scores normed within age (ie, wave) (Fisher *r*-to-*z* tests: *r*_diff_ = 0.07-0.13) (*p* values <.001). Moreover, developmentally scaled scores of the construct-valid items showed the strongest criterion validity (compliance: *r* = −0.25; attention to task: *r* = −0.28). Developmentally scaled scores of the construct-valid items showed significantly stronger criterion validity than the common items and upward/downward extension approaches and *T* and *z* scores (*r*_diff_ = 0.10-0.17; *p* values <.001). Compared to traditional scoring of the construct-valid items, developmental scaling showed stronger criterion validity in relation to the child’s attention to task (*r*_diff_ = 0.04; *z* = −2.25, *p* = .025); however, there was no significant difference in relation to the child’s compliance (*r*_diff_ = 0.03; *z* = −1.29, *p* = .199).

### Incremental Validity

Results of the Bayesian mixed-effects models for evaluating incremental validity are provided in Table 3. Developmentally scaled scores of externalizing problems predicted observations of the child’s compliance and attention to task over and above the following: (1) the common items, (2) items from the upward/downward extension approach, (3) age and sex norm-referenced *T* scores, and (4) *z* scores normed within age. The reverse was not true: the common items, items from the upward/downward extension approach, and *T* and *z* scores did not show incremental validity above the developmentally scaled scores of the construct-valid items.

**Table 3:** Bayesian Mixed-Effect Models

Model	Predictor	Outcome: Attention to task				
		β	SE	Lower	Upper	Significantly greater
1	Common items	0.06	0.03	0.00	0.11	
1	Developmentally scaled construct-valid items	−0.24	0.04	−0.30	−0.17	X
						
2	All possible items (upward/downward extension)	0.15	0.04	0.07	0.23	
2	Developmentally scaled construct-valid items	−0.37	0.05	−0.46	−0.28	X
						
3	*T* scores (age and sex norm-referenced)	0.13	0.03	0.07	0.19	
3	Developmentally scaled construct-valid items	−0.29	0.04	−0.36	−0.22	X
						
4	*z* Scores (normed within age)	0.13	0.03	0.07	0.18	
4	Developmentally scaled construct-valid items	−0.29	0.04	−0.36	−0.22	X

Model	Predictor	Outcome: Compliance				
		β	SE	Lower	Upper	Significantly Greater
5	Common items	0.04	0.03	−0.02	0.09	
5	Developmentally scaled construct-valid items	−0.18	0.04	−0.25	−0.11	X
						
6	All possible items (upward/downward extension)	0.15	0.04	0.07	0.23	
6	Developmentally scaled construct-valid items	−0.30	0.05	−0.40	−0.20	X
						
7	*T* scores (age and sex norm-referenced)	0.12	0.03	0.06	0.18	
7	Developmentally scaled construct-valid items	−0.24	0.04	−0.31	−0.17	X
						
8	*z* Scores (normed within age)	0.10	0.03	0.04	0.16	
8	Developmentally scaled construct-valid items	−0.22	0.04	−0.30	−0.15	X

Note: “Lower” and “Upper” represent the bounds of the 95% credible interval. Beta coefficients represent standardized regression coefficients. Developmentally scaled construct-valid items represent significant terms in the expected direction such that externalizing problem ratings for the child are negatively associated with researchers’ observation of the child’s compliance and attention to task (ie, greater scores on ratings of externalizing problems are associated with poorer compliance and attention to task). “Significantly greater” indicates whether a predictor showed a significantly stronger beta than the other predictor in the model.

## Discussion

Our approach to developmental scaling placed scores from different measures of psychopathology across ages and raters onto the same scale (ie, the construct-valid items approach). All forms of psychopathology decreased across 3 to 7 years of age, for both boys and girls; however, girls showed steeper declines than boys, particularly for externalizing and thought-disordered problems. The most accurate approach, in terms of criterion validity of estimates of externalizing problems in relation to observations of compliance and attention to task, was the use of different items across ages—that is, the construct-valid items approach. The traditionally scored construct-valid items approach was modestly more accurate (*r*_diff_ = 0.07-0.13) than the 2 most widely used traditionally scored approaches to studying people’s trajectories—the common items and the upward/downward extension approaches. Developmental scaling of the construct-valid items—to put the different measures onto the same scale—led to the greatest criterion validity, consistent with the idea that it was the most accurate approach. Developmentally scaled scores showed modestly stronger criterion validity than traditional assessment and scoring approaches (*r*_diff_ = 0.10-0.17). Moreover, developmentally scaled scores showed moderately stronger criterion validity than mean scores of the construct-valid items (*r*_diff_ = 0.03-0.04). In addition, developmentally scaled scores of externalizing problems showed incremental validity above and beyond traditional scoring approaches in predicting observations of noncompliance and inattention. Furthermore, the common items and upward/downward extension approaches led to considerable misclassification of persistence vs desistance with respect to developmental scaling.

Our findings that developmental scaling using age-differing, construct-valid items was the most accurate approach is consistent with theory and findings from simulation work.^7^ The age-related decreases in externalizing problems in early childhood are consistent with prior work.^22^, ^23^, ^24^^,^^41^^,^^42^ The declines in internalizing problems in early childhood are consistent with some prior work,^16^^,^^24^^,^^41^ whereas other studies have identified that internalizing problems show relative stability in level^24^ or even increases^24^^,^^42^, ^43^, ^44^ in early childhood, depending on the rater, for example, parent vs teacher. Studies of thought-disordered problems in community samples are less common; nevertheless, in a sample of young children with autism, girls tended to decrease more in autism symptom severity more than boys,^45^^,^^46^ consistent with our findings.

The study had several limitations. First, the Achenbach scales are potentially less well suited to assess thought-disordered problems than they are to assess externalizing and internalizing problems. Using the *DSM*-oriented Autism Spectrum Problems (CBCL 1.5–5/C–TRF) and the Thought Problems (CBCL 6–18/TRF) subscales, there was only one common item across ages, which may limit the effectiveness of linking thought-disordered scores across development. Moreover, the internal consistency of the thought-disorder items was weak. Second, the sample was a community sample; future work should replicate and extend these findings in a clinical sample. Third, the criterion-related associations with researcher observations were modest, suggesting that researcher observations are an imperfect criterion. Fourth, we observed some systematic missingness, which could lead to altered trajectory estimates such as greater decreases than what might occur normatively. Nevertheless, the developmental courses that we identified are largely consistent with prior work, and we have no reason to expect that this would alter the pattern of associations. Future work should examine additional developmental transitions, such as preadolescence to adolescence.

The study also had key strengths. First, it was innovative; it is the first study to link scores from age- and rater-differing measures of externalizing, internalizing, and thought-disordered problems onto the same scale. Given known rater-role biases^47^—that is, systematic differences in ratings by mothers vs fathers vs teachers, accounting for systematic rater-role biases is a key strength of the study. In addition, it was the first study to empirically compare the construct-valid items approach to traditional measurement approaches. Second, it was longitudinal, allowing one to chart children’s change. Third, it leveraged multiple perspectives of the child’s behavior for more accurate estimates, including ratings by mothers, fathers, and teachers. Fourth, it evaluated the scoring approaches against researcher observations of the child’s behavior, for more objective comparison.

Our study demonstrates the following: (1) that different measures may be necessary to account for heterotypic continuity, and (2) that developmental scaling is an accurate and useful approach to place scores from different measures onto the same scale for charting people’s development and to account for heterotypic continuity. Findings suggest that developmental scaling may enable studying the development of psychopathology across the lifespan. Our work has key implications for assessment and charting people’s development when studying a “moving target,” which applies to many forms of psychopathology. This will lead to more accurate modeling of dimensions of psychopathology and their development. Moreover, researchers may also use this approach to more accurately assess psychological phenomena across cultures.

Our approach also holds promise for better integrating a developmental perspective into the Hierarchical Taxonomy of Psychopathology and the Research Domain Criteria; it allows studying Hierarchical Taxonomy of Psychopathology and Research Domain Criteria dimensions as they manifest in different ways across development, which researchers had not been able to do effectively with traditional approaches.

## CRediT authorship contribution statement

**Isaac T. Petersen:** Writing – review & editing, Writing – original draft, Visualization, Validation, Supervision, Software, Resources, Project administration, Methodology, Investigation, Funding acquisition, Formal analysis, Data curation, Conceptualization. **Zachary Demko:** Writing – review & editing, Visualization, Conceptualization. **Won-Chan Lee:** Writing – review & editing, Methodology, Formal analysis. **Jacob J. Oleson:** Writing – review & editing, Methodology, Formal analysis.
