# Supplementary material for: Studying Development of Psychopathology Using Changing Measures to Account for Heterotypic Continuity
Source: JAACAP Open. 2025 Nov 7;4(1):111–23. doi: 10.1016/j.jaacop.2025.10.008 (PMC12925850; doi:10.1016/j.jaacop.2025.10.008)

Supplement 1. Description of Missing Data.

A total of 230 children had behavior problem ratings from a mother, father, and/or teacher/secondary caregiver; 225 children had scores from researchers' observations of behavior. The number of participants who had data, disaggregated by the number of time points, is in Table S5. Due to the ongoing nature of the longitudinal study, some data are missing because they are not yet available: 14% of participant-by-timepoint instances (i.e., lab visits) are not yet eligible, 4% are eligible and to-be-scheduled, and 2% are eligible and scheduled but not yet conducted. Among eligible participant-by-timepoint instances, 77% of time points had one or more raters provide ratings, and 66% of time points had researchers' observations of behavior. Among missing lab visits at a given time point for which the child reached eligibility, reasons for missingness included: not interested (15%), too busy (15%), moved/relocated (6%), unable to contact (29%), coronavirus (COVID-19) pandemic (28%), and other (6%). Thus, much of the missing instances were due to the COVID-19 pandemic or to not yet being eligible. We suspended lab visits for 14 months during the COVID-19 pandemic (March 2020 – April 2021). We continued to collect online questionnaires from families during the pandemic but were unable to perform lab visits and behavior observations during this period.

We examined whether missingness was systematic in the behavior problem ratings or researchers' behavior observations. In general, older children were more likely to be missing behavior problem ratings (at a trend level; $t[10.48] = -2.14$, $p = .057$) and behavior observations ($t[138.81] = -6.81$, $p < .001$) compared to younger children, presumably due to attrition, which is common in longitudinal studies. Behavior problem ratings ($t[234.42] = 4.97$, $p < .001$) and behavior observations ($t[465.59] = 3.81$, $p < .001$) were also more likely to be missing for those from lower socioeconomic status households. Boys were more likely than girls to be missing

behavior problem ratings (χ^2[1] = 7.67, p = .005) and behavior observations (χ^2[1] = 3.89, p = .049). For behavior problem ratings, compared to Non-Hispanic White participants, ratings were more likely to be missing for Asian (χ^2[1] = 11.47, p < .001), Black (χ^2[1] = 13.85, p < .001), and Hispanic (χ^2[1] = 7.40, p = .007) participants, and participants with some other race (χ^2[1] = 5.90, p = .015), but not for multiracial participants (χ^2[1] = 2.57, p = .109). For behavior observations, compared to Non-Hispanic White participants, ratings were more likely to be missing for Black (χ^2[1] = 5.06, p = .024) and multiracial (χ^2[1] = 5.77, p = .016) participants, but not for Asian (χ^2[1] = 2.77, p = .096), or Hispanic (χ^2[1] = 2.01, p = .156) participants, or for participants with some other race (χ^2[1] = 0.66, p = .416). Compared to participants with behavior problem ratings, participants who were missing behavior problem ratings tended to be higher in observed compliance (t[4.42] = 3.38, p = .024) but did not differ in observed attention to task (t[1.13] = 3.97, p = .134). Compared to participants with behavior observation scores, participants who were missing behavior observations actually tended to be *lower* in externalizing (t[194.26] = 4.50, p < .001), internalizing (t[193.68] = 4.46, p < .001), and thought-disordered (t[192.44] = 5.17, p < .001) problems.

Supplement 2. Developmental Scaling Approach.

We used developmental scaling to link scores from different age-specific measures onto a common latent scale. This allowed meaningful longitudinal comparisons and accurate estimation of psychopathology trajectories. Developmental scaling benefits from having some age-common items from measures at adjacent ages, to serve as an anchor. Developmental scaling can leverage the common items to link the scores from the different measures onto the same scale (i.e., the scale of the reference age). By assuming no changes in functioning of the common items across ages (i.e., no differential item functioning) or by accounting for any changes in item functioning, this approach ensures that both item and latent parameter estimates are expressed on a common scale across ages. Nevertheless, developmental scaling can use all construct-valid items—common and unique items—to estimate people's scores on that scale, thus making use of all construct-valid information while estimating people's scores on a comparable scale across development.

To perform developmental scaling, we used a two-parameter Bayesian longitudinal multidimensional item response model in a mixed modeling item response theory (IRT) framework. Such a model allows us to simultaneously account for heterotypic continuity using different measures across time and to model children's trajectories. The model linked scores from measures across all ages in the same model through simultaneous calibration with a specified reference age. This approach is similar to multiple-group concurrent calibration in test equating and linking[1,2], but it differs in that our longitudinal modeling approach focuses on growth trajectories over time by examining repeated measures of individuals. By incorporating the longitudinal IRT framework, the simultaneous calibration accounts for the within-person dependence of scores over time and is thus more suitable than the group-based concurrent

calibration approach. Moreover, simultaneous calibration results in more precise and stable estimates than separate (two-stage) calibration in which separate models across age are fit.[1,3]

In the longitudinal multidimensional IRT model of the present study, item and person parameter estimates across ages and raters were placed on a common scale using common items and simultaneous calibration. We accounted for age-related differences in item functioning of item parameters (easiness and discrimination), which ensured that the person parameter estimates at different age groups were expressed on the same scale. Rather than estimating parameters separately for each age group, all age-specific measures were calibrated simultaneously within a single model, resulting in item and person parameter estimates on the same underlying scale. To prevent arbitrary shifts in scale, the model imposed an additional constraint: The variance of the latent factor at age 3 was fixed at 1 and the mean was ~0. The scales for subsequent ages were then determined through the common items, preserving score comparability over time.[4,5]

The two-parameter item response model applied intercept–slope parameterization:

$$y_{ij} = \alpha_i \cdot \theta_j + \xi_i \tag{1}$$

where y_{ij} is the score for person j on item i. The model estimates two parameters for each item: easiness (ξ; equivalent to $-1 \times$ difficulty, severity, or threshold) and discrimination (α). The item's easiness parameter is the expected score on an item at a given level of the construct, and is similar to the intercept parameter in factor analysis.[6] The item's discrimination parameter is how strongly the item is associated with the construct, and is similar to a factor loading. Easiness and discrimination provide information about the functioning and usefulness of each item—and the whole measurement scheme—at a given age. In addition, the model estimates a person parameter for each person (i.e., person j): theta (θ). The person parameter, theta, represents a person's level on the latent construct and is similar to a factor score.

A two-parameter logistic IRT model takes the following form:

$$P(y_{ij} = 1|\theta_j, \alpha_i, \xi_i) = \frac{e^{\alpha_i(\theta_j+\xi_i)}}{1+e^{\alpha_i(\theta_j+\xi_i)}} \quad (2)$$

where y_{ij} is the score for person j on item i, theta (θ_j) is the level on the construct for person j, xi (ξ_i) is the easiness parameter for item i, and alpha (α_i) is the discrimination parameter for item i.

For robust estimates of the child's level on each psychopathology dimension, we fit multidimensional item response models that included items assessing the three primary dimensions of psychopathology: externalizing problems, internalizing problems, and thought-disordered problems. This allowed borrowing information from each dimension in the estimation of the other, for more accurate estimates given considerable covariation between externalizing, internalizing, and thought-disordered problems.[7]

In the present study, behavior problem items were rated on a three-point scale that ranged from y_{ij} = 0–2. There were three possible response options (0, 1, 2), so there were two category boundaries: one boundary between 0 and 1, and one boundary between 1 and 2. Because the response options were ordinal, we fit a graded response model, which allows ordinal responses. We used a cumulative response distribution with a logit link. A two-parameter graded response model takes the following general form of Equation (3):

$$P(Y_{ij} = y_{ij}|\theta_j) = P^*_{y_{ij}}(\theta_j) - P^*_{y_{ij}+1}(\theta_j) \quad (3)$$

where:

$$P^*_{y_{ij}}(\theta_j) = P(Y_{ij} \geq y_{ij}|\theta_j, \alpha_i, \xi_{ic}) = \frac{e^{\alpha_i(\theta_j+\xi_{ic})}}{1+e^{\alpha_i(\theta_j+\xi_{ic})}} \quad (4)$$

where y_{ij} is the score for person j on item i, theta (θ_j) is the level on the construct for person j, xi (ξ_{ic}) is the easiness parameter for item i for category c, and alpha (α_i) is the discrimination

parameter for item i.

A cumulative ordinal logistic model involves two separate logistic regression models. The two logistic regressions have different intercepts (easiness) but share the same slopes (discrimination, theta). The first logistic model is outcome 0 versus 1 and 2. The second logistic model is outcome 0 and 1 versus 2. The regression coefficients are on the log odds scale and can be exponentiated for the odds ratio. As an item's easiness increases, the likelihood of endorsing the item increases. As a person's theta increases, the person's level on the construct increases, and thus, their likelihood of endorsing the item increases. In a cumulative ordinal model with a logit link, an item's discrimination parameter represents the increase in log-odds of endorsing a higher response category (e.g., from 0 to 1 or from 1 to 2) for a one-unit increase in a person's level on the latent construct (theta). For instance, if an item's discrimination parameter (α_i) is 1, then a one-unit increase in theta multiplies the odds of endorsing a higher category by exp(1) ≈ 2.72. As an item's discrimination increases, the item becomes more sensitive to differences in theta, meaning that small differences in the person's level on the construct lead to larger differences in the probability of endorsing the item. For instance, if an item's easiness parameter (ξ_{ic}) is -1, the item's discrimination parameter (α_i) is 2, and the person's level on the construct (θ_j) is 0.5, the linear predictor that gets passed to the logit link function is (based on Equation 1):

$$0 = (2 \times 0.5) + (-1)$$

In this case, when passed through the logit link, the probability of endorsing the item (i.e., endorsing 1 or 2 instead of 0, or endorsing 2 instead of 0 or 1) would be 0.5:

$$0.5 = \frac{1}{1+e^{-x}} = \frac{1}{1+e^{-0}} = \frac{1}{1+1}$$

We accounted for potential differential item functioning across ages and raters to ensure we were measuring the same construct across development in a comparable way. We estimated

the item's easiness parameter (ξ_{ic}) with fixed effects for the role of the rater (mother, father, or secondary caregiver), linear and quadratic terms for the child's age, and an age × role interaction. The rater role was dummy coded so that the mother rater was the reference group. The model included a random intercept and random slope for each item. The random slopes for each item were age, role, and an age × role interaction. This allowed each item to differ in its change in easiness over time for each rater type.

We estimated the item's discrimination parameter (α_i) with fixed effects for the psychopathology dimension assessed (externalizing, internalizing, or thought-disordered problems), the role of the rater, linear and quadratic terms for the child's age, and an age × role interaction. The model included a random intercept and random slope for each item. The random slopes for each item were age, rater role, and an age × role interaction. This allowed each item to differ in its change in discrimination over time for each rater type.

We performed the developmental scaling and estimation of growth curves in the same model. Growth curves were estimated for the person parameter (theta; θ_j), representing the child's level on the latent factor for a given dimension of psychopathology (externalizing, internalizing, or thought-disordered problems). A given child had up to four time points. Thus, a quadratic was the most complex polynomial of nonlinear growth we could estimate for children's trajectories that still allow measurement error. Because of prior work demonstrating that developmental trajectories of psychopathology are nonlinear[8], we modeled children's growth with a quadratic term. We modeled random intercepts and random linear and quadratic slopes to allow each child to differ in their starting point, form of growth, and curvature. Age in years was centered to set the intercepts (i.e., reference age) at age 3, the youngest age in the sample. Age 3, therefore, serves as the reference scale, with parameter estimates for subsequent

time points modeled relative to this baseline. Thus, the person and item parameters were on the same scale and were scaled relative to the parameters at 3 years of age. A theta of zero thus represents the average latent level of behavior problems at age 3; a positive theta indicates that the child is above the average latent level of behavior problems at age 3; a negative theta indicates that the child is below the average latent level of behavior problems at age 3.

We included the child's sex (female = 1, male = 0) and the rater role as a predictor of the intercepts and slopes. The fixed effect predictors of the intercepts were the child's age (linear and quadratic terms), rater role, dimension, dimension × role interaction, sex, and a sex × dimension interaction. The fixed effect predictors of the linear slopes were the rater role, dimension, dimension × role interaction, sex, and a sex × dimension interaction. This allowed evaluating sex-related differences in trajectories as a function of the psychopathology dimension. The random slopes for each person were age, quadratic age, role, age × role interaction, dimension, age × dimension interaction, dimension × role interaction, and an age × dimension × role interaction. This allowed each person to have a unique trajectory for each psychopathology dimension and rater type.

In a Bayesian model, the final step is to specify prior distributions for all remaining parameters in the model. The person parameters' (theta) variance was fixed to 1 for model identification and the lower bound of the discrimination parameter to zero, following recommendations for identifiability.[6,9] With two exceptions (described in the previous sentence), we kept the default priors used in the brms package[10], which uses vague but proper priors. The default priors for regression parameters were multivariate normal with mean zero and unknown covariance matrix Σ which follows a LKJ-correlation prior.[11] All standard deviation parameters were given a half Student-*t*-distribution prior with 3 degrees of freedom, mean 0, and scale

parameter 2.5. The prior for the intercept of item discrimination, item easiness, and theta was a flat prior.

Our model had no missing data in the predictors (age, sex, and rater); missingness was only in the outcome (scores on psychopathology items). Mixed models handle missing data in the outcomes. Mixed models provide valid inferences if the data are missing at random or completely at random.[12] Furthermore, our Bayesian hierarchical mixed model also provides valid inference when data are missing at random or completely at random. Because much of our missingness was due to the coronavirus 2019 (COVID-19) pandemic, we felt this modeling approach was appropriate. Moreover, researchers have argued against using multiple imputation in longitudinal designs that use mixed models because multiple imputation can lead to unstable estimates.[13]

Developmentally scaled factor scores were estimated from the posterior distribution by averaging model-predicted posterior samples across chains and iterations, using the posterior_epred() function from the brms package. Model-predicted posterior samples were averaged within combinations of child-by-measurement occasion-by-rater. This allowed each child to have a different factor score of externalizing, internalizing, and thought-disordered problems for each rater at each of their measurement occasions.

We fit the Bayesian longitudinal mixed models using the brm() function of the brms package 2.22[10] in R, which uses the RStan 2.32.6[14] interface to Stan 2.32.2[15] for Bayesian modeling. The models included four chains and 4,000 iterations.

Supplement 3. Latent Class Growth Analyses.

Method

A key goal for developmental psychopathology is to understand the factors that lead to persistence versus desistance of behavior problems. However, to identify processes that influence such developmental courses, it is first necessary to accurately establish which children persist versus desist. As described, there are many problems of using the common items and upward/downward extension approaches to assess children's development of behavior problems. Thus, it is important to determine the extent of misclassification of persistence and desistance that the common items and upward/downward extension approaches would yield with respect to more developmentally sensitive approaches. To determine the extent of misclassification of persistence and desistance that the common items and upward/downward extension approaches yield with respect to developmental scaling, we applied latent class growth analysis using each scoring approach.

Latent class growth analysis models were estimated in Mplus version 8.6[16]. All models used maximum likelihood estimation with robust standard errors (MLR) to account for the nonnormally distributed data. We fit separate latent class growth models for each behavior problem: externalizing, internalizing, and thought-disordered problems.

To estimate latent class growth trajectories given the multiple informants, we estimated a multi-informant composite for that behavior problem at each age based on mothers', fathers', and teachers'/secondary caregivers' reports as indicators. We constrained each informant to have a loading of 1.0 on the multi-informant composite for that behavior problem to (a) keep the composite on an interpretable metric across ages, (b) so as not to give undue weight to some informants than others (given the shared context of mothers and fathers), and (c) given findings

that simpler examination of information from multiple informants tends to do just as well if not better than more complex latent variables.[17] Then, using children's trajectories on the multi-informant composite for that behavior problem, we estimated the intercept, linear slope, and quadratic slope for each latent class. We estimated quadratic growth curves to allow for curvature. Quadratic was the most complex function form we could fit to each child's trajectory (while still accounting for measurement error) because each child had up to four timepoints. We compared the fit criteria and interpretability for one, two, three, and four-class models. Then, for fairness of comparison to the developmentally scaled trajectories, we estimated latent class growth analysis models with the same number of classes for the other scoring approaches: the common items approach and the upward/downward extension approach (which were available, by design, for externalizing problems only). For each model, we determined which class each child belonged to based on the class with which they had the highest assigned probability of membership.

Results

Fit criteria for the one, two, three, and four-class models are in Table S9. In general, as is common in studies using latent class growth analysis, model fit tended to improve as the number of classes increased. However, there appeared to be diminishing returns in fit as the number of classes increased. With three classes, the smallest class for all behavior problem outcomes was small (< 15%). Moreover, the classes tended to represent high, medium, and low trajectories, suggesting dimensional rather than categorical differences. Thus, models with three (or four) trajectories were not particularly interpretable given our goals. Given the modest sample size and our goals of evaluating persistence versus desistance, we selected the two-class models for interpretability. The two-class latent class growth trajectories for externalizing, internalizing, and

thought-disordered problems are in Figure S3. For fairness of comparison to the developmentally scaled trajectories, we estimated two-class models for the other scoring approaches: the common items approach and the upward/downward extension approach (for externalizing problems only).

For externalizing problems, the two-class model of developmentally scaled scores showed a large class that was high and stable/increasing (79.2%; “persisting”) and a second class that was lower and decreasing (20.8%; “desisting”). Using the common items approach, there was a large class that was high (78.7%; “persisting”) and a second class that was lower (21.3%; “low”). With respect to the classifications from the developmental scaling approach, the common items approach misclassified nearly 40% of children: 18.7% were misclassified as persisting and 20.9% were misclassified as low. Using the upward/downward extension approach, both classes were indistinguishable and increased. Thus, the upward/downward extension approach was particularly problematic in terms of accuracy for characterizing children’s trajectories.

For internalizing problems, the two-class model of developmentally scaled scores showed a large class that was high and decreasing (63.8%; “high”) and a second class that was lower and decreasing (36.2%; “low”). Using the common items approach, both classes were indistinguishable and decreased.

For thought-disordered problems, the two-class model of developmentally scaled scores showed a large class that was high and stable/decreasing (53.9%; “high”) and a second class that was lower and decreasing (46.1%; “desisting”). There were not enough age-common items of thought-disordered problems to examine their trajectories in a latent class growth analysis.

Some of the classification discrepancies between the scoring approaches could reflect different percentages of children assigned to each class (i.e., different cutoffs). Nevertheless, the latent class growth analysis demonstrated that the traditional scoring approaches were unable to

characterize the children's trajectories in ways that were consistent with those obtained from the developmental scaling approach. In sum, the common items and upward/downward extension approaches led to considerable misclassification of persistence versus desistance with respect to developmental scaling.

Supplement 4. Sensitivity Analyses.

As a sensitivity analysis, we fit an additional developmental scaling model with longitudinal measurement invariance constraints. In frequentist approaches, measurement invariance is often tested in a four-step approach (configural, scalar, metric, and residual invariance). In a Bayesian approach, however, a conditional logic structure is preferred to sequential model testing because the models provide the information necessary to evaluate the extent of any non-invariance. The advantage of the conditional formulation of Bayesian mixed models is well described in many excellent textbooks[18-20] and the documentation for the R package brms[10]. Wikle et al.[21] (p. 10) wrote: “If most of the complex dependencies in the data are due to the underlying process of interest, then one should model the distribution of the data conditioned on that process (data model), followed by a model of the process’ behavior and its uncertainties (process model).” Therefore, within a Bayesian mixed modeling framework, approximate measurement invariance is a process of interest which can be used to account for small instances of non-invariance.[22,23] Approximate measurement invariance involves setting narrow priors on the invariance parameters rather than fixing invariance parameters to zero.[22] Approximate measurement invariance is more accurate than full or partial measurement invariance for estimating true latent mean differences when there are many small differences in the intercepts and factor loadings across groups.[22,24] Thus, as a sensitivity analysis in the present study, we also fit a model that imposes approximate longitudinal measurement invariance.

In this model, we set the slopes of the discrimination parameters to be close to zero, by setting the prior of the discrimination parameter and easiness parameter to have a normal distribution with a mean of zero and a small standard deviation of 0.05. We also set the prior of the standard deviation of the random effect of item on the association of age with discrimination

and easiness to be small (normal distribution with mean = 0, *SD* = 0.01) so that items were restricted to be similar in their change of discrimination and easiness (i.e., near zero). This approximate measurement invariance approach successfully constrained the slopes of the discrimination and easiness parameters to be near-zero. None of the items showed significant differences in easiness (i.e., intercepts) across ages in this model. Several items showed reliable differences in discrimination (i.e., factor loadings) across ages, but differences were small.

Supplement 5. Item Functioning.

Item Severity and Discrimination

Item parameters by age and rater are in Table S7. For externalizing problems, items that were most strongly associated with the construct (i.e., had the highest discrimination) included items assessing the extent to which the child destroys others' things, has temper tantrums, and is physically aggressive toward others. Items that were more severe (i.e., had a lower easiness) included items assessing the extent to which the child is cruel to animals, is physically aggressive towards others, runs away from home, sets fires, and uses substances. Items that were less severe (i.e., had a higher easiness) included items assessing the extent to which the child argues, wants or demands attention, does not comply at home, and is stubborn.

For internalizing problems, items that were most strongly associated with the construct included items assessing the extent to which the child worries, has rapid changes in moods, feels guilt, and is withdrawn from others. Items that were more severe included items assessing the extent to which the child has somatic issues (e.g., headaches, nausea, vomiting, eye-related problems without a known medical cause) and talks about killing themself. Items that were less severe included items assessing the extent to which the child whines, is clingy to adults, has nightmares, is shy, and has their feelings hurt easily.

For thought-disordered problems, items that were most strongly associated with the construct included items assessing the extent to which the child shows strange behavior, little affection, repetitive behavior, and has hallucinations. Items that were more severe included items assessing the extent to which the child repeatedly rocks their head or body, has hallucinations, and plays with their own genitals in public. Items that were less severe included items assessing the extent to which the child avoids eye contact, has difficulty when things are out of place, and

does not answer when others talk to them.

Changes in Item Functioning With Age

Estimates of changes in item functioning with age are in Table S8. There were several general patterns. First, items tended to increase in easiness with age, suggesting that as children get older, for many of the items, a lower level of the construct is required for the item to be endorsed. For instance, an item assessing the extent to which the child has difficulty sitting still showed increases in easiness with age. Perhaps the increases in item easiness reflect that such behaviors become less developmentally appropriate with age. Consequently, caregivers and teachers may be more concerned or attentive to these behaviors as children age, and when the behavior occurs, it may be more likely to be perceived as a problem. In addition, situational demands increase across the transition from preschool to school entry; for instance, with greater classroom structure, the problematic behaviors may become more visible.

Second, items tended to decrease in discrimination with age, suggesting that some of the behaviors wane in construct relevance. For instance, an item assessing the extent to which the child screams a great deal showed decreases in discrimination with age. It is possible that screaming is a canonical form of dysregulated behavior in younger children, whereas as children get older, they may develop more advanced capacities for expressing their frustration such as arguing or sarcasm that replace the need for screaming.

Third, most of the behaviors showed changes in item functioning, consistent with the notion that psychopathology demonstrates heterotypic continuity. Items that do not show changes in item functioning (e.g., stealing, deviant peers, noncompliance at school) could be valuable anchor items for future longitudinal studies and models. The present study accounted for differential item functioning in the Bayesian item response model.

References

1. Kolen MJ, Brennan RL. *Test equating, scaling, and linking: Methods and practices*. 3rd ed. Statistics for Social and Behavioral Sciences. Springer; 2014:566.
2. Lee W-C, Lee G. IRT linking and equating. *The Wiley Handbook of Psychometric Testing*. 2018:639–673.
3. McArdle JJ, Grimm KJ, Hamagami F, Bowles RP, Meredith W. Modeling life-span growth curves of cognition using longitudinal data with multiple samples and changing scales of measurement. *Psychol Methods*. 2009;14(2):126–149. doi: 10.1037/a0015857
4. Wang C, Nydick SW. On longitudinal item response theory models: A didactic. *J Educ Behav Stat*. 2020;45(3):339–368. doi: 10.3102/1076998619882026
5. Huang H-Y. A multilevel higher order item response theory model for measuring latent growth in longitudinal data. *Appl Psychol Meas*. 2015;39(5):362–372. doi: 10.1177/0146621614568112
6. Bürkner P-C. Bayesian item response modeling in R with brms and Stan. *J Stat Softw*. 2021;100(5):1–54. doi: 10.18637/jss.v100.i05
7. Caspi A, Houts RM, Belsky DW, et al. The p factor: One general psychopathology factor in the structure of psychiatric disorders? *Clin Psychol Sci*. 2014;2(2):119–137. doi: 10.1177/2167702613497473
8. Petersen IT, Bates JE, Dodge KA, Lansford JE, Pettit GS. Describing and predicting developmental profiles of externalizing problems from childhood to adulthood. *Dev Psychopathol*. 2015;27(3):791–818. doi: 10.1017/S0954579414000789
9. Bürkner P-C. Analysing standard progressive matrices (SPM-LS) with Bayesian item response models. *J Intell*. 2020;8(1):5. doi: 10.3390/jintelligence8010005
10. Bürkner P-C. brms: An R package for Bayesian multilevel models using Stan. *J Stat Softw*. 2017;80(1):28. doi: 10.18637/jss.v080.i01
11. Lewandowski D, Kurowicka D, Joe H. Generating random correlation matrices based on vines and extended onion method. *J Multivar Anal*. 2009;100(9):1989–2001. doi: 10.1016/j.jmva.2009.04.008
12. Detry MA, Ma Y. Analyzing repeated measurements using mixed models. *JAMA*. 2016;315(4):407–408. doi: 10.1001/jama.2015.19394
13. Twisk J, de Boer M, de Vente W, Heymans M. Multiple imputation of missing values was not necessary before performing a longitudinal mixed-model analysis. *J Clin Epidemiol*. 2013;66(9):1022–1028. doi: 10.1016/j.jclinepi.2013.03.017
14. *RStan: the R interface to Stan*. 2020. http://mc-stan.org
15. *Stan modeling language users guide and reference manual*. 2020. https://mc-stan.org
16. *Mplus version 8.6*. Muthén & Muthén; 2021. https://www.statmodel.com/programs.shtml
17. Aitken M, Plamondon A, Krzeczkowski J, Kil H, Andrade BF. Systematic integration of multi-informant externalizing ratings in clinical settings. *Res Child Adolesc Psychopathol*. 2023;doi: 10.1007/s10802-023-01119-z
18. Cowles MK. *Applied Bayesian statistics: With R and OpenBUGS examples*. Springer; 2013.
19. Gelman A, Carlin JB, Stern HS, Dunson DB, Vehtari A, Rubin DB. *Bayesian data analysis*. 3rd ed. Taylor & Francis; 2013.
20. Kruschke J. *Doing Bayesian Data Analysis: A Tutorial with R, JAGS, and Stan*. Elsevier Science; 2014.

21. Wikle CK, Zammit-Mangion A, Cressie N. *Spatio-temporal statistics with R*. CRC Press; 2019.
22. Van De Schoot R, Kluytmans A, Tummers L, Lugtig P, Hox J, Muthen B. Facing off with Scylla and Charybdis: a comparison of scalar, partial, and the novel possibility of approximate measurement invariance. *Front Psychol*. 2013;4(770)doi: 10.3389/fpsyg.2013.00770
23. Van De Schoot R, Schmidt P, De Beuckelaer A, Lek K, Zondervan-Zwijnenburg M. Editorial: Measurement invariance. *Front Psychol*. 2015;6(1064)doi: 10.3389/fpsyg.2015.01064
24. Cieciuch J, Davidov E, Schmidt P, Algesheimer R, Schwartz SH. Comparing results of an exact vs. an approximate (Bayesian) measurement invariance test: a cross-country illustration with a scale to measure 19 human values. *Front Psychol*. 2014;5(982)doi: 10.3389/fpsyg.2014.00982

Table S1: Internal Consistency of Behavior Problem Ratings

	Measure							
	CBCL 1.5–5		CBCL 6–18		C–TRF		TRF	
Construct	α	$\omega_{hierarchical}$	α	$\omega_{hierarchical}$	α	$\omega_{hierarchical}$	α	$\omega_{hierarchical}$
Externalizing Problems	.91	.91	.87	.89	.94	.93	.93	–
Internalizing Problems	.82	.80	.80	.80	.84	.81	.78	.76
Thought-Disordered Problems	.62	.63	.50	.51	.70	.38	.47	.47

Note: All α = Cronbach's alpha; $\omega_{hierarchical}$ = omega hierarchical; "–" indicates that the omega coefficient was unable to be estimated due to a model convergence error.

Table S2: Cross-Rater Reliability (Pearson Correlation Coefficients)

		Rater		
	**Externalizing**			
		Mother	Father	Teacher
Rater	Mother	–		
	Father	.52	–	
	Teacher	.42	.40	–
	**Internalizing**			
		Mother	Father	Teacher
Rater	Mother	–		
	Father	.40	–	
	Teacher	.25	.18	–
	**Thought-Disordered**			
		Mother	Father	Teacher
Rater	Mother	–		
	Father	.32	–	
	Teacher	.39	.25	–

Note: All *p*s < .01.

Table S3: 9-Month Lag Cross-Time Rank-Order Stability (Pearson Correlation Coefficients)

Construct	Rater	Cross-Time Stability
Externalizing	Mother	.64
Externalizing	Father	.64
Externalizing	Teacher	.53
Internalizing	Mother	.67
Internalizing	Father	.67
Internalizing	Teacher	.36
Thought-Disordered	Mother	.53
Thought-Disordered	Father	.42
Thought-Disordered	Teacher	.40

Note: All *p*s < .001.

Table S4: Descriptive Statistics and Correlation Matrix of Study Variables

Variable	Age	Sex	SES	EXT (DS)	INT (DS)	TD (DS)	EXT (CV)	EXT (Common)	EXT (All)	Attention (obs)	Compliance (obs)
Age	–										
Sex	.06*	–									
SES	.14***	.06***	–								
EXT (DS)	−.35***	−.09***	−.17***	–							
INT (DS)	−.30***	.03	−.13***	.83***	–						
TD (DS)	−.36***	−.10***	−.16***	.82***	.94***	–					
EXT (CV)	−.26***	−.12***	−.15***	.72***	.56***	.54***	–				
EXT (Common)	−.08***	−.05†	−.12***	.66***	.50***	.46***	.89***	–			
EXT (All)	.02	−.08*	−.13***	.74***	.64***	.59***	.90***	.92***	–		
Attention (obs)	.50***	.14***	.24***	−.28***	−.19***	−.23***	−.24***	−.16***	−.11***	–	
Compliance (obs)	.49***	.12***	.21***	−.24***	−.16***	−.18***	−.22***	−.15***	−.09*	.91***	–
observations	1,857	2,772	2,748	1,328	1,322	1,290	1,328	1,327	981	1,557	1,557
M	4.96	0.47	−0.16	−0.77	−1.63	−2.05	0.15	0.14	0.15	3.93	4.08
SD	1.19	0.50	0.90	2.67	2.86	2.96	0.14	0.15	0.13	0.96	0.99
minimum	2.92	0.00	−3.18	−19.70	−21.71	−24.01	0.00	0.00	0.00	1.00	1.00
maximum	7.80	1.00	2.20	7.49	6.92	6.21	0.77	0.89	0.76	5.00	5.00

Note: "SES" = socioeconomic status (derived from averaging *z*-scores of the parents' educational attainment, the parent's occupational prestige, and of the log transform of the family's income-to-needs ratio); "EXT" = externalizing problems; "INT" = internalizing problems; "TD" = thought-disordered problems; "DS" = developmentally scaled; "CV" = construct-valid items (proportion score); "Common" = common items (proportion score); "All" = all possible items (from the upward/downward extension approach; proportion score); "obs" = observation by researchers. Data are in long form such that each row represents a unique combination of child, wave, and rater. Each participant could have up to 12 rows: 4 timepoints × 3 raters (mother, father, and teacher). Data for the child's sex and socioeconomic status at later timepoints are determined using the last observation carried forward, thus accounting for the larger number of observations. Only parents reported on all possible items (i.e., the upward/downward extension approach), whereas teachers (and parents) reported on the common items and construct-valid items, thus accounting for the relatively smaller number of observations for the externalizing score that leverages all possible items.

Table S5: Number of Participants who had Data by Number of Time Points

	Number of Time Points With Ratings				
Variable	0	1	2	3	4
Behavior Problems: Any Rater	1	49	62	42	77
Behavior Problems: Mother-Report	6	47	62	44	72
Behavior Problems: Father-Report	56	57	61	28	29
Behavior Problems: Teacher/Secondary Caregiver-Report	55	65	59	31	21
Behavior Observation	6	66	58	67	34

Table S6: Regression Coefficients from Bayesian Item Response Model

Parameter	Estimate	*SE*	Lower	Upper
intercept[1]	−1.58	2.26	−6.06	2.83
intercept[2]	1.59	2.26	−2.91	6.01
Predicting Theta				
age (centered)	0.23	0.22	−0.20	0.67
age (centered) quadratic	−0.33	0.09	−0.51	−0.16
mother	0.70	0.46	−0.20	1.62
father	0.43	0.52	−0.56	1.47
secondary caregiver	0.43	0.38	−0.26	1.24
internalizing	−1.34	0.51	−2.29	−0.30
thought disorder	−1.63	0.75	−3.08	−0.12
female	−0.33	0.21	−0.73	0.08
age (centered) × father	0.25	0.15	−0.04	0.55
age (centered) × secondary caregiver	0.19	0.19	−0.21	0.57
age (centered) × internalizing	0.05	0.20	−0.35	0.44
age (centered) × thought disorder	0.11	0.27	−0.44	0.62
father × internalizing	−0.27	0.29	−0.84	0.28
secondary caregiver × internalizing	0.71	0.34	0.02	1.37
father × thought disorder	−0.16	0.37	−0.90	0.56
secondary caregiver × thought disorder	1.30	0.48	0.36	2.25
female × internalizing	0.39	0.21	−0.02	0.79
female × thought disorder	0.44	0.27	−0.10	0.98
age (centered) × female	−0.34	0.17	−0.67	−0.01
age (centered) × father × internalizing	−0.07	0.16	−0.39	0.25
age (centered) × secondary caregiver × internalizing	0.25	0.19	−0.12	0.63
age (centered) × father × thought disorder	0.09	0.21	−0.33	0.51
age (centered) × secondary caregiver × thought disorder	0.12	0.25	−0.38	0.62
age (centered) × female × internalizing	0.05	0.13	−0.21	0.31
age (centered) × female × thought disorder	−0.39	0.17	−0.73	−0.05
Predicting Item Easiness				
intercept	−4.39	2.30	−9.03	0.20
age (centered)	0.41	0.17	0.09	0.75
age (centered) quadratic	−0.02	0.03	−0.07	0.03
father	0.34	0.24	−0.12	0.83
secondary caregiver	−0.58	0.41	−1.40	0.18
age (centered) × father	−0.13	0.08	−0.29	0.02
age (centered) × secondary caregiver	−0.08	0.18	−0.43	0.28

Predicting Item Discrimination				
intercept	0.35	0.04	0.28	0.43
age (centered)	0.00	0.00	0.00	0.01
age (centered) quadratic	0.00	0.00	0.00	0.00
father	0.01	0.01	0.00	0.03
secondary caregiver	0.43	0.10	0.23	0.63
internalizing	0.01	0.01	0.00	0.05
thought disorder	0.03	0.03	0.00	0.10
age (centered) × father	0.00	0.00	0.00	0.01
age (centered) × secondary caregiver	0.05	0.04	0.00	0.15

Note: Regression coefficients are unstandardized. “Lower” and “Upper” represent the bounds of the 95% credible interval. "intercept[1]" represents the intercept for the threshold from 0 to 1; "intercept[2]" represents the intercept for the threshold from 1 to 2. Mother-report is the reference group for the rater role (mother, father, teacher/secondary caregiver). Male is the reference group for the child’s sex. Externalizing is the reference group for the psychopathology dimension (externalizing, internalizing, thought disorder).

Table S7: Item Easiness and Discrimination by Age and Rater

						Easiness						Discrimination					
						Age 3			Age 7.5			Age 3			Age 7.5		
Scale	Construct	CBCL 1.5–5	C–TRF	CBCL 6–18	TRF	M	F	T	M	F	T	M	F	T	M	F	T
EXT	EXT	5	5			−2.87	−2.51	−3.21				1.00	0.94	1.44			
EXT	EXT	6	6			−2.44	−1.90	−2.99				1.04	1.01	1.53			
EXT	EXT	56	56			−4.34	−4.08	−4.69				0.46	0.45	0.75			
EXT	EXT	59	59			−1.50	−1.19	−2.05				0.76	0.73	1.11			
EXT	EXT	95	95			−4.48	−4.21	−4.84				0.88	0.87	1.35			
EXT	EXT	8	8			−1.47	−0.97	−2.61				1.04	1.04	1.65			
EXT	EXT	15	15		6	−2.68	−2.11	−3.56			−2.64	1.17	1.12	1.82			1.54
EXT	EXT	16	16		77	−2.04	−1.62	−3.14			−3.54	1.09	1.07	1.71			1.38
EXT	EXT	18	18	21	21	−4.58	−4.24	−5.25	−2.89	−3.17	−4.15	1.23	1.21	1.91	0.33	0.32	1.60
EXT	EXT	20	20			−2.33	−1.95	−3.17				0.97	0.94	1.63			
EXT	EXT	27	27	26	26	−3.35	−2.82	−3.69	−3.07	−3.09	−3.12	1.04	1.01	1.75	0.21	0.17	1.33
EXT	EXT	29	29			−2.47	−2.14	−3.16				1.12	1.07	1.69			
EXT	EXT	35	35	37	37	−5.64	−5.28	−5.98	−4.70	−4.94	−4.97	1.11	1.08	1.92	0.36	0.33	1.70
EXT	EXT	40	40			−2.94	−2.67	−3.67				1.01	0.96	1.57			
EXT	EXT	42	42			−4.86	−4.55	−5.52				1.05	0.99	1.64			
EXT	EXT	44	44			−3.56	−3.22	−4.20				1.14	1.11	1.80			
EXT	EXT	53	53	57	57	−5.47	−5.19	−6.03	−4.04	−4.36	−5.16	1.15	1.10	1.86	0.39	0.35	1.58
EXT	EXT	58	58			−3.52	−3.14	−4.11				1.23	1.22	1.92			
EXT	EXT	66	66	68	68	−3.73	−3.21	−4.52	−2.85	−2.87	−4.57	1.23	1.19	1.76	0.47	0.46	1.43
EXT	EXT	69	69			−2.74	−2.31	−3.24				0.77	0.77	1.45			
EXT	EXT	81	81	86	86	−3.02	−2.68	−3.55	−0.61	−0.89	−2.19	1.16	1.14	1.72	0.21	0.19	1.30
EXT	EXT	85	85	95	95	−2.65	−2.32	−3.84	−0.87	−1.14	−3.58	1.25	1.22	1.92	0.38	0.36	1.64
EXT	EXT	88	88			−3.26	−3.02	−4.02				1.13	1.09	1.67			
EXT	EXT	96	96			−1.98	−1.66	−2.81				0.93	0.92	1.21			

EXT	EXT		2				−6.02	−6.28				0.20	0.18	
EXT	EXT		28	28			−1.11	−1.36	−2.66			0.41	0.39	1.17
EXT	EXT		39	39			−3.99	−4.12	−4.35			0.29	0.29	0.87
EXT	EXT		43	43			−1.65	−1.84	−2.81			0.36	0.37	0.90
EXT	EXT		63	63			−1.80	−1.93	−3.74			0.20	0.23	0.40
EXT	EXT		67				−7.44	−7.75				0.34	0.36	
EXT	EXT		72				−7.41	−7.72				0.34	0.35	
EXT	EXT		73				−7.44	−7.75				0.34	0.35	
EXT	EXT		81				−4.33	−4.69				0.42	0.41	
EXT	EXT		82	82			−4.99	−5.24	−5.41			0.36	0.35	1.40
EXT	EXT		90	90			−3.87	−4.07	−4.83			0.46	0.48	1.18
EXT	EXT		96	96			−6.77	−7.04	−7.09			0.26	0.26	0.80
EXT	EXT		99	99			−7.68	−7.99	−7.70			0.34	0.36	0.90
EXT	EXT		101	101			−6.27	−6.59	−6.77			0.30	0.32	0.81
EXT	EXT		105	105			−7.66	−7.96	−7.67			0.34	0.36	0.90
EXT	EXT		106				−5.42	−5.72				0.35	0.39	
EXT	EXT		3	3			0.54	0.30	−1.60			0.32	0.32	1.14
EXT	EXT	22	16	16		−5.25	−3.09	−3.47	−3.25		1.80	0.31	0.30	1.53
EXT	EXT		19	19			0.15	−0.05	−1.65			0.45	0.45	1.14
EXT	EXT	17	20	20		−5.24	−2.71	−2.99	−4.27		1.27	0.35	0.35	1.37
EXT	EXT		22				−0.46	−0.70				0.36	0.38	
EXT	EXT		23	23			−2.75	−2.96	−2.95			0.24	0.21	1.10
EXT	EXT		87	87			−1.45	−1.79	−3.07			0.44	0.43	0.94
EXT	EXT		88	88			−2.98	−3.26	−3.07			0.35	0.35	0.94
EXT	EXT		89	89			−5.91	−6.22	−5.84			0.49	0.51	1.48
EXT	EXT	84	94	94		−6.02	−2.62	−3.07	−3.41		1.16	0.34	0.35	1.27
EXT	EXT		97	97			−4.39	−4.66	−5.77			0.39	0.41	0.90
EXT	EXT		104	104			−2.57	−2.75	−4.23			0.55	0.59	1.48
EXT	EXT	24				−2.90					1.38			

EXT	EXT		48					−3.41						1.05			
EXT	EXT		51					−3.77						0.83			
EXT	EXT		64					−3.20						1.16			
EXT	EXT		14					−6.62						0.75			
EXT	EXT		28					−4.02						1.41			
EXT	EXT		74					−6.31						1.05			
EXT	EXT				98						−4.98						0.83
EXT	EXT				76						−4.22						1.44
INT	INT	21	21			−2.69	−2.20	−3.62				0.56	0.60	0.98			
INT	INT	46	46			−6.37	−6.10	−6.57				0.59	0.61	1.03			
INT	INT	51				−5.95	−5.67					1.26	1.21				
INT	INT	79				−3.97	−3.71					0.99	0.97				
INT	INT	82	82			−3.13	−2.83	−3.87				1.00	0.98	1.55			
INT	INT	83	83			−4.50	−4.26	−4.76				0.64	0.65	1.14			
INT	INT	92	92			−4.49	−4.12	−5.26				0.86	0.83	1.27			
INT	INT	97	97			−0.97	−0.46	−2.39				0.50	0.49	1.04			
INT	INT	99	99	112	112	−3.96	−3.81	−4.59	−1.01	−1.53	−2.34	0.53	0.53	0.94	0.35	0.37	1.04
INT	INT	10	10			−1.73	−1.07	−2.90				0.64	0.64	1.04			
INT	INT	33	33			−1.94	−1.68	−2.38				0.51	0.52	0.90			
INT	INT	37	37			−2.67	−2.20	−3.42				0.70	0.71	1.14			
INT	INT	43	43			−5.50	−5.30	−5.69				0.61	0.60	1.14			
INT	INT	47	47	45	45	−4.75	−4.56	−5.27	−1.67	−2.12	−2.88	0.96	0.91	1.32	0.42	0.38	1.16
INT	INT	68	68	71	71	−3.28	−3.02	−3.80	−0.64	−1.03	−1.42	0.45	0.45	0.82	0.24	0.25	0.79
INT	INT	87	87	50	50	−4.64	−4.27	−5.42	−1.95	−2.17	−3.48	0.75	0.75	1.07	0.48	0.52	1.16
INT	INT	90	90	103	103	−5.99	−5.78	−6.25	−3.68	−4.10	−3.68	0.67	0.68	1.26	0.32	0.33	1.21
INT	INT	1	1	56a	56a	−4.05	−3.68	−4.88	−3.61	−3.82	−5.05	0.24	0.26	0.71	0.30	0.31	0.99
INT	INT	7	7			−2.59	−2.11	−3.45				0.44	0.46	0.73			
INT	INT	12		49		−3.00	−2.69		−2.45	−2.74		0.28	0.29		0.33	0.34	
INT	INT	19				−4.60	−4.22					0.38	0.37				

INT	INT	24				−2.52	−2.14					0.57	0.56				
INT	INT	39	39	56b	56b	−6.48	−6.30	−6.88	−2.93	−3.43	−3.60	0.43	0.43	0.83	0.13	0.13	0.66
INT	INT	45	45	56c	56c	−6.41	−6.12	−6.67	−3.73	−4.08	−3.84	0.44	0.46	0.95	0.48	0.51	1.26
INT	INT	52				−4.51	−4.19					0.18	0.22				
INT	INT	78	78	56f	56f	−5.25	−4.96	−5.81	−3.31	−3.66	−3.93	0.32	0.34	0.76	0.29	0.32	0.88
INT	INT	86	86			−3.79	−3.47	−4.59				0.48	0.45	0.76			
INT	INT	93	93	56g	56g	−6.17	−5.80	−6.89	−4.96	−5.18	−6.48	0.28	0.29	0.63	0.45	0.48	0.96
INT	INT	2	2			−3.57	−3.10	−3.91				0.52	0.50	1.07			
INT	INT	4	4			−3.16	−2.69	−3.64				0.53	0.52	1.11			
INT	INT	23	23			−1.88	−1.42	−2.43				0.67	0.62	1.30			
INT	INT	62	62			−4.59	−4.16	−5.55				0.75	0.72	1.28			
INT	INT	67	67			−6.74	−6.47	−7.00				1.17	1.13	1.88			
INT	INT	70	70			−5.42	−5.05	−5.52				0.71	0.71	1.41			
INT	INT	71	71			−5.53	−5.17	−5.85				0.64	0.63	1.28			
INT	INT	98	98	111	111	−5.31	−4.94	−5.67	−4.36	−4.58	−4.78	0.93	0.92	1.58	0.37	0.36	1.42
INT	INT			14	14				−1.59	−1.71	−2.78				0.33	0.36	0.94
INT	INT			29	29				−2.06	−2.21	−4.62				0.25	0.26	0.70
INT	INT			30	30				−3.66	−3.92	−4.67				0.24	0.23	0.91
INT	INT			31	31				−3.15	−3.26	−4.21				0.39	0.41	1.19
INT	INT			32	32				−1.19	−1.50	−1.80				0.36	0.36	0.69
INT	INT			33	33				−2.48	−2.68	−4.29				0.10	0.05	1.03
INT	INT			35	35				−3.64	−3.87	−5.62				0.38	0.38	1.08
INT	INT			52	52				−3.97	−4.13	−5.95				0.45	0.48	1.09
INT	INT			91	91				−5.15	−5.40	−6.35				0.42	0.43	1.14
INT	INT			5	5				−4.33	−4.73	−3.38				0.21	0.18	1.17
INT	INT			42	42				−2.69	−2.85	−3.22				0.26	0.25	0.76
INT	INT			65	65				−3.73	−3.87	−4.90				0.26	0.27	0.82
INT	INT			69	69				−3.06	−3.37	−3.71				0.29	0.30	0.85
INT	INT			75	75				−1.32	−1.52	−2.57				0.19	0.23	0.55

INT	INT			102	102				−4.32	−4.68	−3.83				0.31	0.33	0.58
INT	INT			47					−1.36	−1.67					0.22	0.25	
INT	INT			51	51				−5.16	−5.42	−6.17				0.39	0.43	0.78
INT	INT			54	54				−4.28	−4.64	−4.68				0.35	0.37	1.01
INT	INT			56d	56d				−6.13	−6.34	−6.77				0.31	0.32	0.80
INT	INT			56e	56e				−2.67	−2.85	−4.48				0.24	0.20	0.41
INT	INT		12					−5.24						1.02			
INT	INT		19					−3.32						0.70			
INT	INT				81						−2.03						0.63
INT	INT				106						−3.86						0.57
INT	INT				108						−2.20						0.63
DSM	TD	4	4			−3.07	−2.63	−3.68				0.61	0.60	1.13			
DSM	TD	7	7			−2.48	−2.04	−3.42				0.48	0.50	0.64			
DSM	TD	21	21			−2.56	−2.13	−3.56				0.57	0.59	0.83			
DSM	TD	23	23			−1.74	−1.35	−2.42				0.71	0.66	1.21			
DSM	TD	25	25			−4.08	−3.81	−4.48				0.58	0.56	1.06			
DSM	TD	63	63			−6.44	−6.14	−6.81				0.66	0.67	1.23			
DSM	TD	67	67			−6.78	−6.50	−7.21				1.23	1.19	1.90			
DSM	TD	70	70			−5.45	−5.08	−5.69				0.85	0.84	1.49			
DSM	TD	76	76			−3.36	−3.04	−3.73				0.39	0.41	0.96			
DSM	TD	80	80	84	84	−6.63	−6.40	−6.75	−5.09	−5.48	−4.31	0.87	0.85	1.24	0.39	0.39	1.05
DSM	TD	92	92			−4.28	−3.93	−5.13				0.78	0.76	1.05			
DSM	TD	98	98			−5.36	−5.02	−5.83				0.95	0.94	1.47			
TP	TD			9	9				−0.68	−0.97	−1.74				0.54	0.56	1.12
TP	TD			18	18				−6.33	−6.64	−6.81				0.37	0.39	0.93
TP	TD			40	40				−7.29	−7.58	−7.46				0.39	0.41	0.96
TP	TD			46	46				−4.23	−4.47	−5.22				0.40	0.42	0.78
TP	TD			58	58				−1.51	−1.62	−3.40				0.18	0.20	0.54
TP	TD			59					−5.33	−5.53					0.39	0.40	

TP	TD	60			−4.32	−4.56			0.17	0.20	
TP	TD	66	66		−3.75	−4.00	−4.21		0.43	0.44	1.16
TP	TD	70	70		−5.81	−6.11	−6.54		0.40	0.42	0.97
TP	TD	76			−2.01	−2.36			0.28	0.32	
TP	TD	83	83		−2.58	−2.79	−4.96		0.34	0.34	0.86
TP	TD	85	85		−5.74	−5.99	−6.21		0.38	0.39	1.11
TP	TD	92			−3.56	−3.95			0.15	0.17	
TP	TD	100			−2.85	−3.29			0.33	0.36	

Note: “EXT” = Externalizing; “INT” = Internalizing; “TD” = Thought Disorder; “DSM” = DSM Autism Spectrum; “TP” = Thought Problems. Yellow highlighted cells represent items that span across the age-differing measures. Gray highlighted cells indicates items that were not included in the Bayesian item response model for that rater at a given age (based on the items that are intended to be assessed by each rater on the Achenbach System of Empirically Based Assessment), consistent with the construct-valid items approach. Some of the items are included in multiple scales; for instance, item 46 of the CBCL 6–18 is part of the Thought Problems (Thought Disorder) subscale, whereas it is part of the Internalizing scale of the CBCL 1.5–5 and C–TRF. In addition, items 4, 7, 21, 23, 67, 70, 92, 98 of the of the CBCL 1.5–5 and C–TRF are in the DSM Autism Spectrum (Thought Disorder) subscale, whereas they are also part of the Internalizing scale of the CBCL 1.5–5 and C–TRF. For these items, they were allowed to load onto each of the relevant factors at the relevant ages as part of the Bayesian item response model. For example, item 4 was allowed to load onto both the Thought Disorder and Internalizing factors at ages 3–5.

Table S8: Estimates of Differential Item Functioning by Age

		Slope of Easiness				Slope of Discrimination			
Construct	Item Number	Estimate	Error (*SD*)	Lower	Upper	Estimate	Error (*SD*)	Lower	Upper
Externalizing	CBCL 1.5–5: 15	**0.58**	0.20	0.19	0.97	**−0.18**	0.05	−0.28	−0.09
Externalizing	CBCL 1.5–5: 16	**0.47**	0.19	0.10	0.85	**−0.17**	0.05	−0.26	−0.07
Externalizing	CBCL 1.5–5: 18	**0.47**	0.20	0.08	0.85	**−0.20**	0.04	−0.29	−0.12
Externalizing	CBCL 1.5–5: 20	**0.49**	0.19	0.13	0.87	**−0.15**	0.05	−0.24	−0.07
Externalizing	CBCL 1.5–5: 27	0.15	0.18	−0.20	0.52	**−0.19**	0.04	−0.26	−0.11
Externalizing	CBCL 1.5–5: 29	**0.75**	0.20	0.37	1.16	**−0.18**	0.05	−0.28	−0.09
Externalizing	CBCL 1.5–5: 35	0.30	0.21	−0.10	0.72	**−0.17**	0.05	−0.27	−0.06
Externalizing	CBCL 1.5–5: 40	0.25	0.20	−0.12	0.65	**−0.16**	0.05	−0.26	−0.07
Externalizing	CBCL 1.5–5: 42	0.29	0.22	−0.13	0.73	**−0.17**	0.06	−0.28	−0.06
Externalizing	CBCL 1.5–5: 44	**0.58**	0.21	0.18	1.00	**−0.19**	0.05	−0.29	−0.09
Externalizing	CBCL 1.5–5: 5	**0.56**	0.19	0.19	0.97	**−0.17**	0.05	−0.27	−0.08
Externalizing	CBCL 1.5–5: 53	**0.41**	0.21	0.01	0.82	**−0.17**	0.05	−0.27	−0.07
Externalizing	CBCL 1.5–5: 56	0.36	0.19	0.00	0.73	−0.04	0.05	−0.15	0.06
Externalizing	CBCL 1.5–5: 58	**0.41**	0.21	0.00	0.84	**−0.20**	0.05	−0.31	−0.10
Externalizing	CBCL 1.5–5: 59	0.24	0.17	−0.09	0.58	**−0.11**	0.04	−0.20	−0.03
Externalizing	CBCL 1.5–5: 6	**0.71**	0.20	0.33	1.11	**−0.19**	0.05	−0.29	−0.10
Externalizing	CBCL 1.5–5: 66	0.29	0.20	−0.09	0.69	**−0.17**	0.04	−0.25	−0.09
Externalizing	CBCL 1.5–5: 69	0.30	0.18	−0.04	0.65	**−0.09**	0.05	−0.19	0.00
Externalizing	CBCL 1.5–5: 8	**0.68**	0.19	0.31	1.06	**−0.17**	0.05	−0.26	−0.08
Externalizing	CBCL 1.5–5: 81	**0.63**	0.19	0.27	1.00	**−0.21**	0.03	−0.27	−0.15
Externalizing	CBCL 1.5–5: 85	**0.49**	0.19	0.12	0.86	**−0.20**	0.03	−0.26	−0.13
Externalizing	CBCL 1.5–5: 88	**0.54**	0.21	0.15	0.95	**−0.17**	0.05	−0.27	−0.07
Externalizing	CBCL 1.5–5: 95	0.13	0.21	−0.28	0.57	**−0.11**	0.06	−0.22	−0.01
Externalizing	CBCL 1.5–5: 96	**0.53**	0.18	0.18	0.89	**−0.15**	0.05	−0.24	−0.05
Externalizing	CBCL 6–18: 101	0.23	0.29	−0.35	0.82	0.02	0.11	−0.20	0.22

Externalizing	CBCL 6–18: 104	0.47	0.26	−0.03	0.98	0.00	0.09	−0.17	0.18
Externalizing	CBCL 6–18: 105	0.12	0.31	−0.50	0.72	0.01	0.11	−0.20	0.23
Externalizing	CBCL 6–18: 16	**0.51**	0.23	0.08	0.96	**−0.16**	0.07	−0.29	−0.03
Externalizing	CBCL 6–18: 19	**0.72**	0.24	0.26	1.22	−0.07	0.07	−0.20	0.06
Externalizing	CBCL 6–18: 20	**0.48**	0.22	0.06	0.91	−0.07	0.06	−0.20	0.05
Externalizing	CBCL 6–18: 23	0.39	0.25	−0.10	0.88	−0.08	0.07	−0.22	0.07
Externalizing	CBCL 6–18: 28	**0.54**	0.24	0.07	1.01	−0.03	0.07	−0.16	0.10
Externalizing	CBCL 6–18: 3	**0.63**	0.23	0.18	1.10	−0.09	0.06	−0.20	0.02
Externalizing	CBCL 6–18: 39	0.34	0.26	−0.16	0.87	−0.03	0.09	−0.21	0.14
Externalizing	CBCL 6–18: 43	**0.60**	0.24	0.13	1.09	0.03	0.07	−0.10	0.17
Externalizing	CBCL 6–18: 63	0.33	0.24	−0.14	0.80	−0.03	0.06	−0.15	0.10
Externalizing	CBCL 6–18: 82	0.26	0.27	−0.27	0.80	−0.11	0.10	−0.31	0.08
Externalizing	CBCL 6–18: 87	**0.65**	0.24	0.18	1.13	−0.01	0.07	−0.15	0.14
Externalizing	CBCL 6–18: 88	0.42	0.25	−0.05	0.92	−0.01	0.08	−0.17	0.15
Externalizing	CBCL 6–18: 89	0.30	0.29	−0.27	0.86	−0.02	0.11	−0.23	0.19
Externalizing	CBCL 6–18: 90	0.27	0.26	−0.24	0.79	0.00	0.09	−0.17	0.18
Externalizing	CBCL 6–18: 94	**0.74**	0.23	0.30	1.20	−0.05	0.07	−0.18	0.08
Externalizing	CBCL 6–18: 96	0.18	0.30	−0.42	0.77	−0.01	0.11	−0.23	0.21
Externalizing	CBCL 6–18: 97	0.40	0.28	−0.14	0.96	0.02	0.10	−0.17	0.21
Externalizing	CBCL 6–18: 99	0.12	0.31	−0.49	0.74	0.01	0.11	−0.21	0.24
Externalizing	CBCL 6–18: 106	0.33	0.29	−0.22	0.90	0.08	0.11	−0.13	0.29
Externalizing	CBCL 6–18: 2	0.27	0.30	−0.31	0.86	−0.05	0.11	−0.27	0.17
Externalizing	CBCL 6–18: 22	**0.55**	0.24	0.09	1.03	−0.03	0.06	−0.16	0.09
Externalizing	CBCL 6–18: 67	0.14	0.31	−0.47	0.77	0.00	0.12	−0.24	0.23
Externalizing	CBCL 6–18: 72	0.15	0.31	−0.45	0.75	0.00	0.11	−0.23	0.22
Externalizing	CBCL 6–18: 73	0.15	0.30	−0.44	0.74	0.00	0.11	−0.22	0.22
Externalizing	CBCL 6–18: 81	0.43	0.28	−0.10	1.00	0.01	0.10	−0.19	0.21
Externalizing	C–TRF: 14	0.22	0.31	−0.38	0.83	0.00	0.09	−0.18	0.18
Externalizing	C–TRF: 24	**0.59**	0.26	0.10	1.09	−0.09	0.07	−0.23	0.06

Externalizing	C–TRF: 28	**0.62**	0.26	0.12	1.14	−0.09	0.08	−0.23	0.07
Externalizing	C–TRF: 48	**0.62**	0.25	0.14	1.14	−0.04	0.07	−0.17	0.11
Externalizing	C–TRF: 51	**0.80**	0.26	0.29	1.33	−0.01	0.07	−0.15	0.13
Externalizing	C–TRF: 64	**0.71**	0.25	0.21	1.20	−0.08	0.07	−0.22	0.06
Externalizing	C–TRF: 74	0.42	0.29	−0.14	1.00	−0.03	0.09	−0.20	0.14
Externalizing	TRF: 76	0.38	0.28	−0.16	0.94	−0.09	0.11	−0.30	0.13
Externalizing	TRF: 98	0.34	0.29	−0.24	0.91	0.03	0.11	−0.19	0.24
Internalizing	CBCL 1.5–5: 1	0.19	0.17	−0.15	0.53	0.01	0.04	−0.06	0.09
Internalizing	CBCL 1.5–5: 10	0.28	0.20	−0.09	0.67	−0.05	0.04	−0.14	0.03
Internalizing	CBCL 1.5–5: 12	0.22	0.17	−0.11	0.55	0.01	0.03	−0.05	0.07
Internalizing	CBCL 1.5–5: 2	**0.52**	0.19	0.15	0.91	−0.05	0.05	−0.14	0.03
Internalizing	CBCL 1.5–5: 21	**0.43**	0.20	0.06	0.83	−0.01	0.05	−0.10	0.08
Internalizing	CBCL 1.5–5: 23	0.22	0.20	−0.15	0.61	−0.08	0.04	−0.17	0.00
Internalizing	CBCL 1.5–5: 33	**0.71**	0.18	0.36	1.08	−0.03	0.04	−0.11	0.04
Internalizing	CBCL 1.5–5: 37	0.00	0.21	−0.41	0.41	**−0.10**	0.05	−0.20	−0.02
Internalizing	CBCL 1.5–5: 39	**0.88**	0.21	0.47	1.31	−0.07	0.05	−0.16	0.03
Internalizing	CBCL 1.5–5: 4	**0.49**	0.19	0.10	0.87	−0.03	0.05	−0.12	0.06
Internalizing	CBCL 1.5–5: 43	**0.59**	0.25	0.13	1.10	−0.05	0.06	−0.17	0.06
Internalizing	CBCL 1.5–5: 45	**0.69**	0.23	0.26	1.14	0.01	0.06	−0.10	0.12
Internalizing	CBCL 1.5–5: 46	**0.65**	0.27	0.14	1.19	−0.03	0.07	−0.16	0.10
Internalizing	CBCL 1.5–5: 47	**0.78**	0.22	0.37	1.21	**−0.12**	0.04	−0.20	−0.04
Internalizing	CBCL 1.5–5: 62	0.41	0.23	−0.03	0.87	**−0.11**	0.05	−0.23	−0.01
Internalizing	CBCL 1.5–5: 67	0.12	0.30	−0.46	0.70	**−0.20**	0.07	−0.34	−0.06
Internalizing	CBCL 1.5–5: 68	**0.68**	0.17	0.35	1.01	−0.05	0.03	−0.10	0.00
Internalizing	CBCL 1.5–5: 7	0.20	0.18	−0.15	0.57	0.00	0.04	−0.08	0.09
Internalizing	CBCL 1.5–5: 70	**0.54**	0.25	0.06	1.05	−0.09	0.06	−0.21	0.03
Internalizing	CBCL 1.5–5: 71	0.33	0.25	−0.15	0.83	−0.07	0.06	−0.19	0.05
Internalizing	CBCL 1.5–5: 78	**0.52**	0.19	0.15	0.91	−0.01	0.04	−0.10	0.08
Internalizing	CBCL 1.5–5: 82	0.44	0.24	−0.01	0.92	**−0.15**	0.05	−0.25	−0.05

Internalizing	CBCL 1.5–5: 83	**0.45**	0.22	0.02	0.88	−0.09	0.05	−0.20	0.01
Internalizing	CBCL 1.5–5: 86	0.10	0.20	−0.28	0.49	−0.05	0.05	−0.15	0.05
Internalizing	CBCL 1.5–5: 87	**0.69**	0.21	0.29	1.11	−0.06	0.04	−0.14	0.02
Internalizing	CBCL 1.5–5: 90	**0.61**	0.22	0.19	1.06	−0.08	0.05	−0.19	0.02
Internalizing	CBCL 1.5–5: 92	0.13	0.25	−0.33	0.63	**−0.12**	0.06	−0.23	−0.01
Internalizing	CBCL 1.5–5: 93	0.36	0.24	−0.08	0.84	0.04	0.06	−0.08	0.15
Internalizing	CBCL 1.5–5: 97	0.22	0.18	−0.12	0.56	−0.03	0.04	−0.10	0.04
Internalizing	CBCL 1.5–5: 98	0.30	0.22	−0.14	0.75	**−0.13**	0.05	−0.23	−0.02
Internalizing	CBCL 1.5–5: 99	**0.75**	0.18	0.39	1.11	−0.04	0.03	−0.10	0.02
Internalizing	CBCL 6–18: 102	0.26	0.27	−0.28	0.78	0.05	0.09	−0.14	0.24
Internalizing	CBCL 6–18: 14	**0.50**	0.25	0.03	1.01	0.01	0.07	−0.12	0.14
Internalizing	CBCL 6–18: 29	0.32	0.25	−0.16	0.82	−0.01	0.07	−0.15	0.12
Internalizing	CBCL 6–18: 30	0.36	0.26	−0.15	0.90	−0.01	0.08	−0.17	0.16
Internalizing	CBCL 6–18: 31	0.27	0.26	−0.22	0.79	−0.05	0.09	−0.22	0.12
Internalizing	CBCL 6–18: 32	**0.54**	0.25	0.07	1.03	−0.06	0.08	−0.21	0.08
Internalizing	CBCL 6–18: 33	**0.52**	0.26	0.03	1.03	**−0.20**	0.07	−0.33	−0.06
Internalizing	CBCL 6–18: 35	0.36	0.27	−0.16	0.90	−0.07	0.09	−0.26	0.12
Internalizing	CBCL 6–18: 42	0.36	0.25	−0.11	0.85	0.01	0.07	−0.13	0.15
Internalizing	CBCL 6–18: 5	**0.58**	0.28	0.03	1.13	−0.11	0.10	−0.32	0.09
Internalizing	CBCL 6–18: 51	0.27	0.28	−0.25	0.85	0.10	0.10	−0.10	0.30
Internalizing	CBCL 6–18: 52	0.15	0.28	−0.39	0.71	−0.02	0.10	−0.21	0.17
Internalizing	CBCL 6–18: 54	0.44	0.28	−0.09	0.99	0.00	0.10	−0.19	0.18
Internalizing	CBCL 6–18: 56d	0.17	0.30	−0.41	0.75	0.03	0.11	−0.18	0.25
Internalizing	CBCL 6–18: 56e	0.36	0.26	−0.13	0.87	0.03	0.07	−0.11	0.17
Internalizing	CBCL 6–18: 65	0.37	0.27	−0.15	0.89	−0.03	0.09	−0.21	0.14
Internalizing	CBCL 6–18: 69	0.50	0.26	−0.01	1.03	−0.03	0.08	−0.20	0.13
Internalizing	CBCL 6–18: 75	**0.54**	0.24	0.07	1.02	−0.02	0.06	−0.13	0.09
Internalizing	CBCL 6–18: 91	0.20	0.28	−0.34	0.76	−0.05	0.10	−0.26	0.16
Internalizing	CBCL 1.5–5: 19	0.00	0.23	−0.45	0.47	−0.02	0.06	−0.13	0.09

Internalizing	CBCL 1.5–5: 24	0.13	0.19	−0.24	0.52	−0.06	0.04	−0.15	0.03
Internalizing	CBCL 1.5–5: 51	0.48	0.29	−0.07	1.05	**−0.21**	0.07	−0.36	−0.08
Internalizing	CBCL 1.5–5: 52	0.36	0.21	−0.05	0.77	0.05	0.05	−0.05	0.16
Internalizing	CBCL 1.5–5: 79	0.36	0.25	−0.12	0.86	**−0.13**	0.06	−0.25	−0.02
Internalizing	CBCL 6–18: 47	**0.52**	0.25	0.03	1.01	0.01	0.06	−0.10	0.13
Internalizing	C–TRF: 12	**0.65**	0.28	0.11	1.19	−0.02	0.08	−0.18	0.14
Internalizing	C–TRF: 19	**0.65**	0.25	0.15	1.15	0.02	0.07	−0.12	0.16
Internalizing	TRF: 106	0.38	0.28	−0.17	0.93	0.07	0.11	−0.14	0.27
Internalizing	TRF: 108	**0.55**	0.27	0.02	1.09	0.06	0.10	−0.14	0.26
Internalizing	TRF: 81	0.51	0.27	−0.01	1.05	0.09	0.10	−0.11	0.29
Thought Disordered	CBCL 1.5–5: 21	**0.47**	0.20	0.07	0.86	−0.02	0.05	−0.12	0.07
Thought Disordered	CBCL 1.5–5: 23	0.29	0.21	−0.12	0.71	−0.08	0.04	−0.17	0.01
Thought Disordered	CBCL 1.5–5: 25	**0.42**	0.21	0.01	0.85	−0.07	0.05	−0.18	0.03
Thought Disordered	CBCL 1.5–5: 4	**0.55**	0.21	0.14	0.96	−0.05	0.05	−0.14	0.04
Thought Disordered	CBCL 1.5–5: 63	0.51	0.27	−0.02	1.06	−0.05	0.07	−0.19	0.10
Thought Disordered	CBCL 1.5–5: 67	0.11	0.30	−0.47	0.70	**−0.20**	0.07	−0.35	−0.06
Thought Disordered	CBCL 1.5–5: 7	0.25	0.20	−0.13	0.63	0.00	0.05	−0.10	0.09
Thought Disordered	CBCL 1.5–5: 70	**0.59**	0.26	0.11	1.10	−0.12	0.06	−0.25	0.00
Thought Disordered	CBCL 1.5–5: 76	0.29	0.19	−0.08	0.65	−0.03	0.05	−0.13	0.06
Thought Disordered	CBCL 1.5–5: 80	0.44	0.25	−0.04	0.94	−0.11	0.07	−0.24	0.02
Thought Disordered	CBCL 1.5–5: 92	0.12	0.24	−0.35	0.60	−0.09	0.06	−0.21	0.03
Thought Disordered	CBCL 1.5–5: 98	**0.55**	0.26	0.04	1.06	**−0.13**	0.06	−0.26	−0.01
Thought Disordered	CBCL 6–18: 18	0.23	0.30	−0.36	0.84	0.01	0.11	−0.21	0.22
Thought Disordered	CBCL 6–18: 40	0.14	0.31	−0.46	0.75	0.01	0.11	−0.20	0.24
Thought Disordered	CBCL 6–18: 46	0.38	0.28	−0.18	0.94	0.04	0.10	−0.16	0.24
Thought Disordered	CBCL 6–18: 58	0.31	0.25	−0.19	0.81	0.00	0.06	−0.11	0.11
Thought Disordered	CBCL 6–18: 66	0.37	0.28	−0.18	0.91	0.00	0.10	−0.19	0.18
Thought Disordered	CBCL 6–18: 70	0.27	0.29	−0.29	0.83	0.00	0.11	−0.21	0.22
Thought Disordered	CBCL 6–18: 83	**0.54**	0.28	0.02	1.10	−0.02	0.09	−0.19	0.18

Thought Disordered CBCL 6–18: 85	0.16	0.29	−0.41	0.74	−0.03	0.11	−0.25	0.19
Thought Disordered CBCL 6–18: 9	**0.83**	0.28	0.30	1.39	0.10	0.08	−0.05	0.28
Thought Disordered CBCL 6–18: 100	0.42	0.27	−0.11	0.97	−0.01	0.09	−0.20	0.17
Thought Disordered CBCL 6–18: 59	0.22	0.29	−0.35	0.81	0.00	0.11	−0.21	0.21
Thought Disordered CBCL 6–18: 60	0.37	0.28	−0.17	0.94	−0.02	0.10	−0.21	0.18
Thought Disordered CBCL 6–18: 76	**0.55**	0.26	0.05	1.10	0.03	0.07	−0.12	0.18
Thought Disordered CBCL 6–18: 92	0.41	0.27	−0.10	0.97	−0.05	0.09	−0.22	0.12

Note: Bolded coefficients represent significant differential item functioning across ages. "*SD*" = standard deviation.

Table S9: Model Fit of Latent Class Growth Analysis Models by Number of Classes

Externalizing Problems

# Classes	LL	AIC	BIC	SABIC	Entropy	LMR-LRT *p*	BLRT *p*	Smallest Class (%)
1	model did not converge				–	–	–	–
2	−2637.953	5373.906	5542.372	5387.071	0.827	.032	< .001	20.8
3	−2508.292	5122.583	5304.801	5136.823	0.815	.181	< .001	14.0
4	−2464.480	5042.961	5238.931	5058.276	0.856	.240	< .001	1.2

Internalizing Problems

# Classes	LL	AIC	BIC	SABIC	Entropy	LMR-LRT *p*	BLRT *p*	Smallest Class (%)
1	model did not converge				–	–	–	–
2	−2784.824	5667.648	5836.114	5680.813	0.679	.240	< .001	36.2
3	−2730.002	5566.004	5748.222	5580.244	0.727	.240	< .001	5.0
4	−2693.881	5501.763	5697.733	5517.078	0.717	.240	< .001	4.7

Thought-Disordered Problems

# Classes	LL	AIC	BIC	SABIC	Entropy	LMR-LRT *p*	BLRT *p*	Smallest Class (%)
1	model did not converge				–	–	–	–
2	−2720.562	5539.125	5707.590	5552.290	0.683	.240	< .001	46.1
3	−2633.318	5372.637	5554.855	5386.877	0.769	< .001	< .001	9.1
4	−2607.703	5329.406	5525.376	5344.721	0.701	.216	< .001	8.7

Note: “LMR-LRT” = Lo-Mendell-Rubin adjusted likelihood ratio test; “BLRT” = bootstrapped likelihood ratio test.

Table S10: Fisher's r-to-z Tests of Comparative Criterion-Related Validity

Correlation 1			Correlation 2					
Variable 1	Variable 2	*r*	Variable 1	Variable 2	*r*	r_{diff}	*z*	*p*
Attention to Task	Construct-Valid Items	−.24	Attention to Task	Common Items	−.16	.08	−5.54	< **.001**
Attention to Task	Construct-Valid Items (Parent-Report Only)	−.22	Attention to Task	All Possible Items (Upward/Downward Extension)	−.12	.10	−7.18	< **.001**
Attention to Task	Construct-Valid Items	−.24	Attention to Task	*T*-Scores (Age and Sex Norm-Referenced)	−.11	.13	−6.39	< **.001**
Attention to Task	Construct-Valid Items	−.24	Attention to Task	*z*-Scores (Normed Within Age)	−.12	.12	−10.30	< **.001**
Attention to Task	Developmentally Scaled Construct-Valid Items	−.28	Attention to Task	Common Items	−.16	.12	−5.22	< **.001**
Attention to Task	Developmentally Scaled Construct-Valid Items (Parent-Report Only)	−.26	Attention to Task	All Possible Items (Upward/Downward Extension)	−.12	.14	−5.76	< **.001**
Attention to Task	Developmentally Scaled Construct-Valid Items	−.28	Attention to Task	*T*-Scores (Age and Sex Norm-Referenced)	−.11	.17	−7.88	< **.001**
Attention to Task	Developmentally Scaled Construct-Valid Items	−.28	Attention to Task	*z*-Scores (Normed Within Age)	−.12	.16	−7.18	< **.001**
Attention to Task	Developmentally Scaled Construct-Valid Items	−.28	Attention to Task	Construct-Valid Items	−.24	.04	−2.25	**.025**
Compliance	Construct-Valid Items	−.22	Compliance	Common Items	−.15	.07	−5.17	< **.001**
Compliance	Construct-Valid Items (Parent-Report Only)	−.20	Compliance	All Possible Items (Upward/Downward Extension)	−.09	.11	−7.50	< **.001**
Compliance	Construct-Valid Items	−.22	Compliance	*T*-Scores (Age and Sex Norm-Referenced)	−.09	.13	−6.78	< **.001**
Compliance	Construct-Valid Items	−.22	Compliance	*z*-Scores (Normed Within Age)	−.11	.11	−10.11	< **.001**
Compliance	Developmentally Scaled Construct-Valid Items	−.25	Compliance	Common Items	−.15	.10	−4.11	< **.001**
Compliance	Developmentally Scaled Construct-Valid Items (Parent-Report Only)	−.25	Compliance	All Possible Items (Upward/Downward Extension)	−.09	.16	−5.39	< **.001**

Compliance	Developmentally Scaled Construct-Valid Items	−.25	Compliance	*T*-Scores (Age and Sex Norm-Referenced)	−.09	.16	−7.26	< **.001**
Compliance	Developmentally Scaled Construct-Valid Items	−.25	Compliance	*z*-Scores (Normed Within Age)	−.11	.14	−6.16	< **.001**
Compliance	Developmentally Scaled Construct-Valid Items	−.25	Compliance	Construct-Valid Items	−.22	.03	−1.29	.199

Note: Bolded *p*-values represent significant differences between correlation 1 and correlation 2 (based on Fisher's *r*-to-*z* test).

Figure S1: Participant Flow Chart

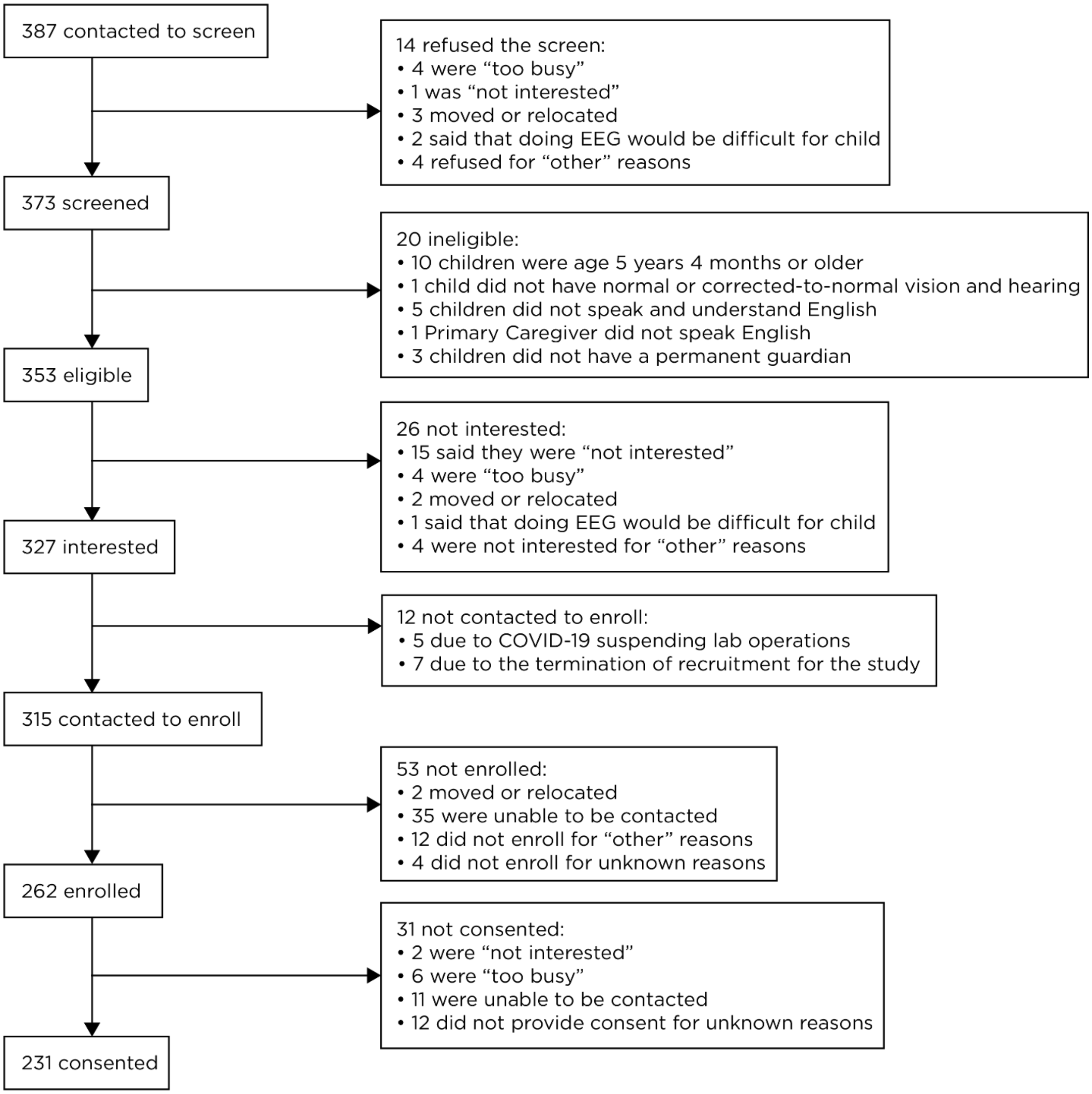

Note: “EEG” = electroencephalography.

Figure S2: Accelerated Longitudinal Design

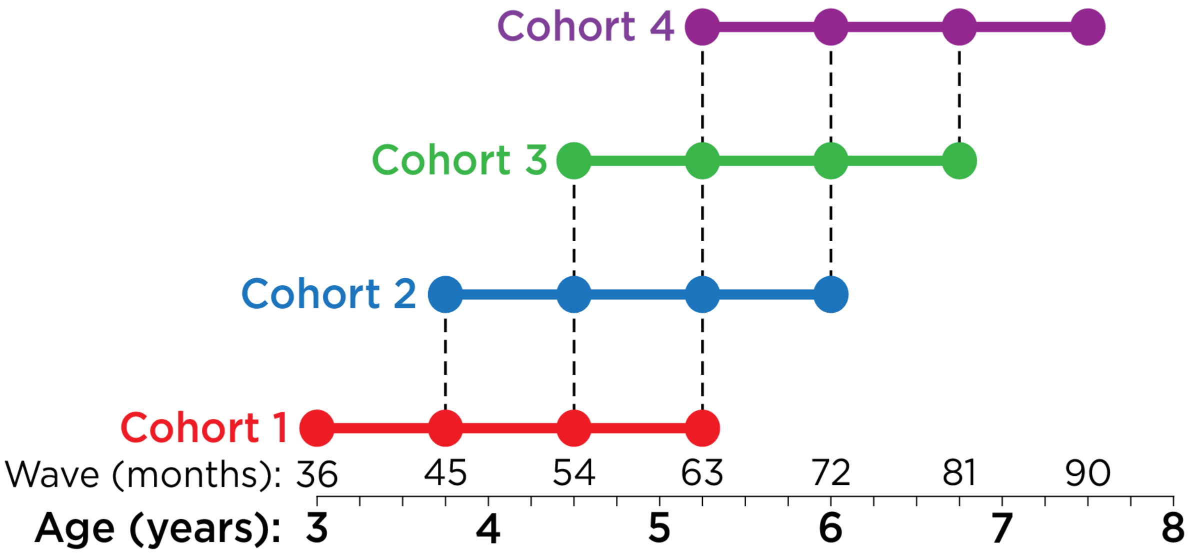

Note: Accelerated longitudinal research design with four cohorts. The longitudinal design follows any given child for 2¼ years, with testing every nine months; the whole data set spans the ages of 3–7½ years. Circles reflect measurement points (four waves) for each cohort. Dashed lines indicate common measurement points across cohorts.

Figure S3: Latent Class Growth Analysis Trajectories

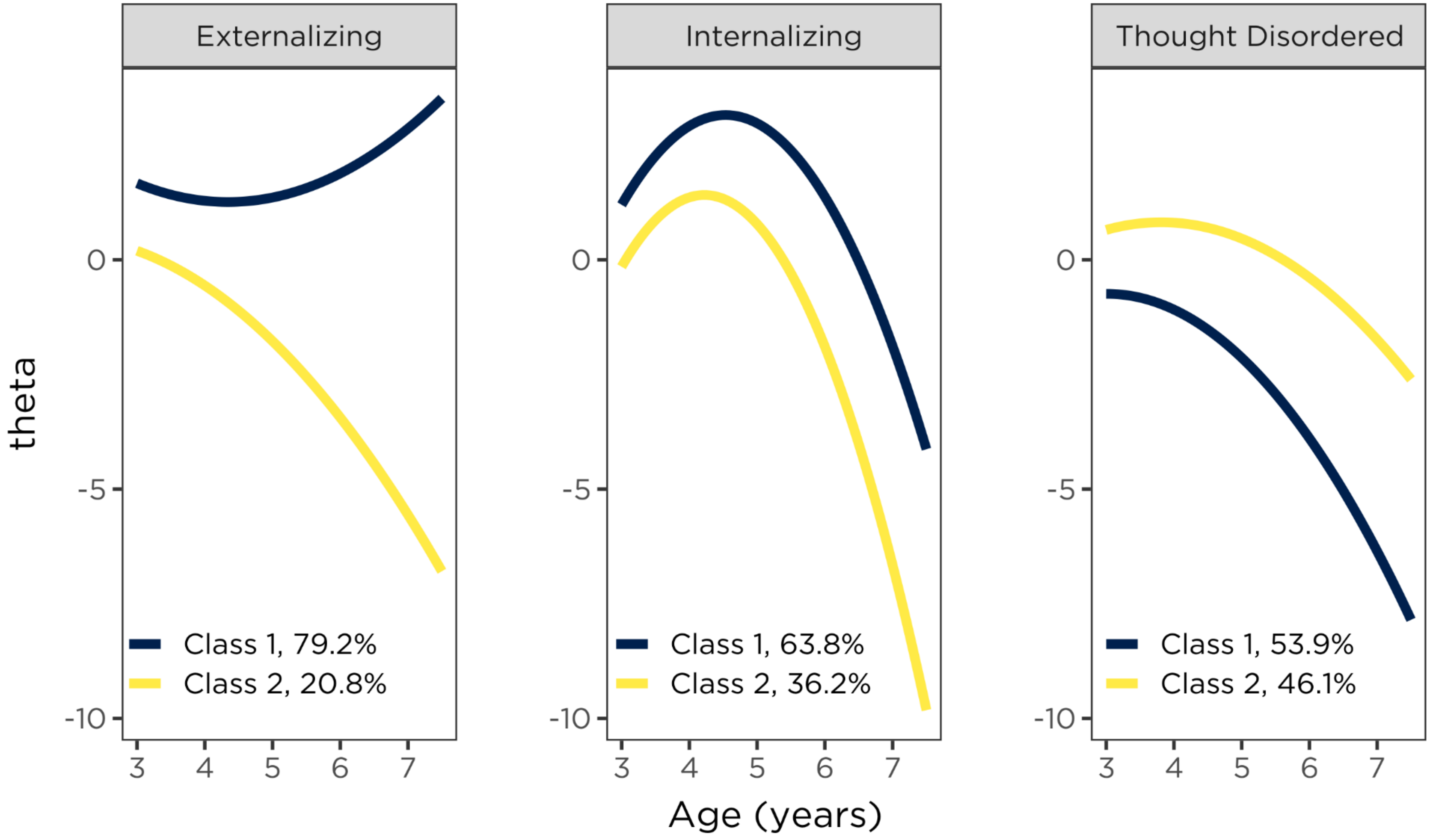

Supplement: Supplementary Material [file mmc1.pdf]
